# Regulatory T Cells and Nanomaterials: Dual Perspectives in Therapeutics and Immunomodulation

**DOI:** 10.1002/smsc.202500481

**Published:** 2025-11-19

**Authors:** Yiyin Chen, Haibo Huang, Xiang Wang, Xinghao Yu, Ziyan Huang, Zhou Jin, Chen Chen, Yan Chen, Bruce R. Blazar, Yang Xu, Yunjie Lu

**Affiliations:** ^1^ The First Affiliated Hospital of Soochow University Soochow University Suzhou 215000 China; ^2^ National Clinical Research Center for Hematologic Diseases Jiangsu Institute of Hematology, The First Affiliated Hospital of Soochow University, Soochow University Suzhou 215000 China; ^3^ Institute of Blood and Marrow Transplantation Collaborative Innovation Center of Hematology Soochow University Suzhou 215000 China; ^4^ Taizhou Second People's Hospital Affiliated to Yangzhou University Taizhou 225300 China; ^5^ Xinghua People's Hospital Affiliated to Yangzhou University Taizhou 225700 China; ^6^ Department of Anesthesiology The First People's Hospital of Changzhou Changzhou 213000 China; ^7^ Department of General Surgery Wujin Affiliated Hospital of Jiangsu University and The Wujin Clinical College of Xuzhou Medical University Changzhou 213000 China; ^8^ Department of Pediatrics Division of Blood & Marrow Transplant & Cellular Therapy University of Minnesota Minneapolis MN 55455 USA

**Keywords:** autoimmunity, cancer immunotherapies, nanoparticles, regulatory T cells, transplant tolerance

## Abstract

Regulatory T cells (Tregs) orchestrate immune tolerance, protecting against autoimmunity and promoting transplant tolerance, yet they can also facilitate tumor immune evasion. Advances in nanotechnology now permit high‐precision manipulation of Treg biology. Tailored polymeric, lipid‐based, inorganic, and biomimetic nanoparticles can be engineered to deliver antigens, cytokines, small‐molecule drugs, antibodies, or nucleic acids that selectively expand or stabilize Tregs for tolerogenic therapy; the same design principles can be inverted to inhibit or deplete intratumoral Tregs, thereby restoring effective antitumor immunity. Beyond intentional therapies, the review also explores unintended immunological consequences of nanoparticles, such as inadvertent induction of Tregs or broader immunosuppressive responses, and how Tregs can conversely limit the efficacy of nanoparticle‐based vaccines or cancer nanotherapies. Outstanding challenges related to targeting efficiency, safety, manufacturability, and combinatorial therapeutic strategies are outlined, and prospective avenues for future investigation are highlighted. Collectively, emerging data position Treg‐focused nanomedicine as a versatile and clinically relevant toolkit for restoring or unleashing immunity across autoimmunity, transplantation, and oncology.

## Graphical abstract

Tregs control immune tolerance but can also enable tumor escape. Nanomaterials can now expand or stabilize Tregs to treat autoimmunity and support transplantation or conversely disrupt tumor Tregs to boost antitumor immunity. We also discuss unintended Treg modulation by nanoparticles, safety and manufacturing challenges, and future translational opportunities.

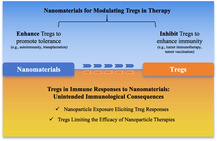

## 1 Introduction

Regulatory T cells (Tregs) are a specialized subset of CD4^+^ T cells that maintain immune self‐tolerance and homeostasis.^[^ ^1^ ^]^ By producing immunoregulatory cytokines (e.g., interleukin‐10 (IL‐10) and transforming growth factor‐β (TGF‐β)) and directly suppressing effector lymphocytes, Tregs prevent pathologic autoimmunity and restrain excessive inflammatory responses.^[^ ^2^ ^]^ However, these same cells can also impede protective immunity: in cancer, tumor‐infiltrating Tregs create a tolerogenic microenvironment that dampens antitumor immune responses.^[^ ^3^ ^]^ Imbalances in Treg activity are therefore implicated in a spectrum of diseases, in which loss or insufficiency of Treg function contributes to autoimmunity and transplant rejection, whereas excessive or mislocalized Treg activity permits cancers and certain chronic infections to evade immune clearance.^[^ ^3^, ^4^ ^]^ This functional duality makes Tregs an attractive target for therapeutic modulation: boosting Treg numbers or function can restore tolerance in autoimmunity, inflammatory disorders, and transplantation, whereas inhibiting or reprogramming Tregs may enhance immunity in cancer and other settings.

Nanomaterials have emerged as powerful tools for precise and context‐dependent manipulation of the immune system.^[^ ^5^ ^]^ Nanoparticles (NPs) can be engineered with defined size, composition, and surface chemistry to deliver drugs, antigens, or genetic materials to selected immune cell populations. Critically, NPs enable cell‐selective targeting and controlled release of immunomodulators, which may reduce the systemic toxicity associated with conventional immunosuppressive or immunostimulatory drugs.^[^ ^6^ ^]^ In the context of Tregs, researchers have designed nanotherapeutics that act as tolerogenic “artificial antigen‐presenting cells (APCs)” to expand Tregs or restore regulatory networks.^[^ ^7^, ^8^ ^]^ Conversely, nanocarriers are being developed to selectively deplete or reprogram Tregs in cancer, where Tregs remain a major barrier to durable antitumor immunity.^[^ ^9^ ^]^ The versatility of nanomaterials (spanning liposomes, polymeric NPs, metallic NPs, etc.) allows them to either enhance or suppress immune functions depending on the payload and targeting ligands used.^[^ ^10^ ^]^

In this review, we examined two complementary aspects of the interplay between nanomaterials and Tregs. First, we discussed the therapeutic use of nanomaterials to modulate Tregs across various disease contexts, including autoimmune and inflammatory diseases, transplantation, and cancer immunotherapy. We highlighted the mechanistic strategies used, including NP‐mediated delivery of tolerogenic signals and targeting of Treg‐specific surface markers. Second, we addressed the roles of Tregs in immune responses to nanomaterials, focusing on how endogenous regulatory pathways respond to NP exposure, whether intentionally engaged by design or triggered unintentionally. These unintended immunological consequences were important for nanomedicine safety and efficacy: while an NP‐induced tolerogenic effect might mitigate inflammatory toxicities, it could also weaken host defenses or reduce the efficacy of vaccines and immunotherapies. Finally, we considered the practical barriers to clinical translation and highlight future opportunities at the intersection of nanotechnology and Treg‐targeted therapy.

## 2 Tregs Ontogeny and Functions

Tregs are a specialized subset of CD4^+^ T lymphocytes that serve as crucial moderators of the immune system. They play an indispensable role in maintaining immunological self‐tolerance and preventing excessive immune reactions that could damage the host.^[^ ^11^ ^]^ In healthy individuals, Tregs typically constitute ≈5–10% of peripheral CD4^+^ T cells.^[^ ^11^ ^]^ The importance of Tregs is underscored by rare disorders like IPEX syndrome, caused by mutations in the Treg‐defining gene *FOXP3*. In IPEX, the absence of functional Tregs leads to rampant autoimmunity and severe multiorgan damage from early infancy, demonstrating that Tregs are essential for immune homeostasis and protection from pathologic autoimmunity.^[^ ^12^ ^]^

Tregs predominantly develop in the thymus as a functionally mature T cell subpopulation during T cell ontogeny.^[^ ^1^ ^]^ Thymus‐derived Tregs (tTregs, also called natural Tregs (nTregs)) arise when developing T cells encounter high‐affinity self‐antigens in the thymus under specific conditions. Their lineage commitment and maturation in the thymus are orchestrated by three key signals: (1) strong T cell receptor (TCR) recognition of self‐peptide‐MHC ligands, (2) costimulatory signaling through CD28 (interacting with CD80/CD86 on thymic APCs), and (3) cytokine signaling from IL‐2 (and in mice also IL‐15).^[^ ^13^, ^14^ ^]^ These signals initiate the Treg lineage program and upregulate the master transcription factor FOXP3.^[^ ^15^, ^16^ ^]^ A subset of self‐reactive thymocytes is thereby diverted from an autoimmune effector fate and differentiates into suppressive tTregs that enforce self‐tolerance.^[^ ^14^, ^17^ ^]^ Beyond the thymus, conventional CD4^+^ T cells can convert to peripherally induced Tregs (pTregs, sometimes called iTregs) when antigen is encountered under tolerogenic conditions that include TGF‐β, IL‐2, and adjunct cues such as retinoic acid, which together induce and stabilize *FOXP3* expression.^[^ ^18^, ^19^ ^]^ pTregs commonly arise at environmental interfaces, particularly at mucosal sites such as the intestine, to maintain tolerance to dietary antigens and commensal microbes. Both tTregs and pTregs depend on IL‐2 for survival and suppressive function, underscoring the centrality of IL‐2 signaling throughout the Treg life course.^[^ ^20^ ^]^ In mouse models, tTregs maintain durable lineage stability, whereas pTregs are more labile and can lose FOXP3, reverting to conventional CD4^+^ T cells. Owing to this stronger fate commitment, tTregs are considered the safer, preferred substrate for adoptive Treg therapy and are being actively advanced for autoimmune and inflammatory indications.^[^ ^21^ ^]^

Tregs maintain immune homeostasis by curbing excessive or misdirected responses across innate and adaptive compartments. They limit activation of conventional CD4^+^ and CD8^+^ T cells, B cells, natural killer cells, and APCs, particularly dendritic cells (DCs), through layered mechanisms that operate in parallel and reinforce one another.^[^ ^1^, ^22^ ^]^ Tregs secrete inhibitory cytokines such as IL‐10, TGF‐β, and IL‐35, which restrain proinflammatory T helper cells and macrophages and condition DCs toward a tolerogenic phenotype.^[^ ^23^ ^]^ They constitutively express the high affinity IL‐2 receptor α chain CD25, allowing efficient capture of IL‐2 and depriving effector T cells of a critical growth factor, thereby limiting clonal expansion by cytokine restriction.^[^ ^20^, ^24^ ^]^ Tregs also express checkpoint receptors, most notably CTLA‐4, which binds CD80 and CD86 on antigen presenting cells with greater affinity than CD28 and reduces costimulatory signaling to other T cells; CTLA‐4 engagement promotes indoleamine‐2,3‐dioxygenase (IDO) activity in DCs and local tryptophan catabolism that suppresses effector function.^[^ ^25^ ^]^ Additional inhibitory receptors, including LAG‐3 and TIGIT, transmit dampening signals upon ligand engagement on antigen presenting or target cells.^[^ ^26^, ^27^ ^]^ Under certain conditions, Tregs deploy cytolytic pathways, releasing perforin and granzyme B to induce apoptosis of activated T cells or NK cells in a contact dependent manner.^[^ ^28^, ^29^, ^30^ ^]^ Tregs also reshape the extracellular metabolic milieu by expressing CD39 and CD73, which convert proinflammatory extracellular ATP into adenosine that inhibits effector lymphocytes through A2A receptors.^[^ ^31^ ^]^ Together, these pathways impose a localized, multipronged brake on immunity and preserve peripheral tolerance in diverse inflammatory settings.

## 3 Nanomaterials for Modulating Tregs in Therapy

**Figure 1:**
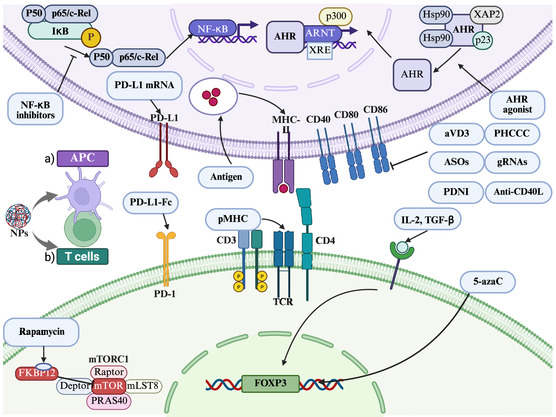
Nanomaterial‐enabled mechanisms for promoting Treg‐mediated immune tolerance in both autoimmune and transplant contexts. The figure is depicted through two complementary strategies: (a) reprogramming of APCs and (b) direct conditioning of T cells. In the first approach, nanomaterials reprogram APCs toward a tolerogenic phenotype. NPs delivering specific autoantigens to APCs promote presentation, augmenting Treg priming. These carriers co‐deliver immunomodulators (aVD3, PHCCC, NF‐κB inhibitors), genetic regulators (ASOs against CD40/CD80/CD86; CRISPR/Cas9 components such as gRNAs to costimulatory pathways), immune‐modulating biologics (anti‐CD40L antibodies, PDNI), and PD‐L1 mRNA. Collectively, these interventions suppress APC activation, downregulate co‐stimulatory ligands, and skew cytokine output toward an anti‐inflammatory profile. Finally, activation of the AhR pathway by NP‐delivered ligands (e.g., ITE) consolidates tolerogenic APC programming: ligand‐bound cytosolic AhR (released from Hsp90/XAP2/p23 complexes) translocates to the nucleus, dimerizes with ARNT and, together with CBP/p300, drives XRE‐dependent transcription of immunoregulatory genes, thereby promoting tolerogenic APC phenotypes. In the second approach, nanomaterials directly condition T cells. Antigen‐specific pMHC displayed by nanomaterials engages the TCR‐CD3 complex on T cells, skewing differentiation toward a regulatory phenotype. Co‐delivery of IL‐2 and TGF‐ via nanoparticles (NPs) activates IL‐2R‐JAK1/3‐STAT5 together with TGF‐βR‐SMAD2/3, thereby inducing and stabilizing *FOXP3*. The DNA methyltransferase inhibitor 5‐azaC, delivered by NPs, reinforces Treg commitment by demethylating CNS2/TSDR at the FOXP3 locus. PD‐L1‐Fc presented by NPs engages PD‐1 on T cells to deliver inhibitory signaling that induces anergy or supports conversion of conventional CD4^+^ T cells into induced Tregs. Additionally, rapamycin‐loaded NPs release rapamycin to inhibit mTORC1, preferentially promoting Treg differentiation. Created in BioRender. Chen, Y. (2025) https://BioRender.com/dg1lyk7. Abbreviations: APCs, antigen‐presenting cells; Treg, regulatory T cell; pMHC, peptide‐MHC; 5‐azaC, 5‐azacytidine; aVD_3_, 1,25‐dihydroxyvitamin D_3_; AhR, aryl hydrocarbon receptor; ARNT, aryl hydrocarbon receptor nuclear translocator; ASO, antisense oligonucleotide; gRNA, guide RNA; Hsp90, heat‐shock protein‐90; IKK, IκB kinase; IL‐2, interleukin‐2; mRNA, messenger RNA; mTORC1, mechanistic target of rapamycin complex‐1; NF‐κB, nuclear factor κB; NP, nanoparticle; PD‐L1, programmed death‐ligand‐1; PDNI, polydopamine nanoparticle; TCR, T‐cell receptor; TGF‐β, transforming growth factor‐β; XRE, xenobiotic response element.

**Figure 2:**
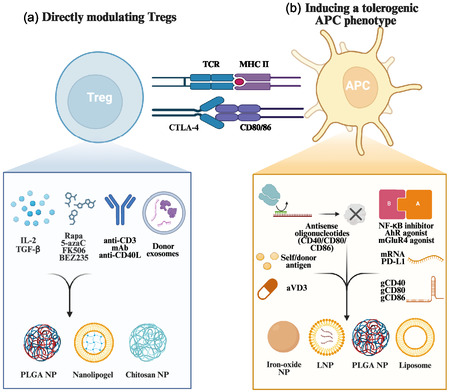
Nanocarrier‐cargo‐target‐cell map for potentiating Treg‐mediated immune tolerance. a) Direct modulation of Tregs. NPs, nanolipogels encapsulating cytokines (e.g., IL‐2 and TGF‐β), small‐molecule inhibitors targeting mTOR/PI3K pathways or epigenetic modulators (rapamycin, FK506, BEZ235, and 5‐azacytidine), monoclonal antibodies that engage the T cell receptor complex (anti‐CD3) or block costimulation (anti‐CD40L), as well as donor‐derived exosomes, directly engage Tregs upon systemic or local administration. This targeted delivery provides essential survival factors or transcriptional signals, enhancing Treg proliferation, phenotypic stability, and immunosuppressive function. b) Induction of tolerogenic APC phenotypes. A complementary strategy uses iron‐oxide NPs, LNPs, PLGA NPs, or liposomes to ferry agents that rewire APCs toward tolerance. Payloads include self or donor antigens, the active vitamin‐D metabolite aVD3, antisense oligonucleotides that silence the costimulatory ligands CD40, CD80, and CD86, immunometabolic or transcriptional modulators such as NF‐κB inhibitors, AhR agonists and mGluR4 agonists, as well as nucleic acids that add or edit inhibitory checkpoints (mRNA‐encoded PD‐L1 or guide RNAs gCD40, gCD80 and gCD86 for CRISPR editing). Once internalized by DCs or macrophages, these nanocarriers down‐tune costimulatory pathways, upregulate tolerogenic signals and promote expansion of Foxp3^+^ Tregs while restraining pathogenic effector responses. Together, the two nanotechnology platforms furnish versatile tools for increasing Treg abundance and function, offering promising therapeutic avenues for autoimmune disorders, inflammatory diseases, and transplantation tolerance. Created in BioRender. Chen, Y. (2025) https://BioRender.com/5plmg03. Abbreviations: aVD3, 1,25‐dihydroxyvitamin D_3_; AhR, aryl hydrocarbon receptor; APC, antigen‐presenting cell; BEZ235, dual PI3K/mTOR inhibitor; CTLA‐4, cytotoxic‐T‐lymphocyte‐associated protein 4; gRNA, guide RNA; IL‐2, interleukin‐2; LNP, lipid nanoparticle; mAb, monoclonal antibody; mGluR4, metabotropic glutamate receptor‐4; mRNA, messenger RNA; NF‐κB, nuclear factor κ‐light‐chain‐enhancer of activated B cells; NP, nanoparticle; PD‐L1, programmed death‐ligand 1; PLGA, poly(lactic‐co‐glycolic acid); Rapa, rapamycin; TCR, T cell receptor; TGF‐β, transforming growth factor‐β; Treg, regulatory T cell.

Therapeutic nanomedicine has leveraged various nanomaterials—including polymeric NPs, liposomes, inorganic nanocarriers, lipid NPs (LNPs), and hybrid constructs—to precisely modulate Treg populations. Different classes of nanomaterials offer unique interactions with the immune system based on their surface properties. For example, polymeric NPs like PLGA can be functionalized with targeting ligands (e.g., antibodies) and provide controlled release of payloads, whereas lipid‐based carriers (liposomes, LNPs) can fuse with cell membranes and incorporate immune‐signaling molecules on their surfaces. Inorganic NPs (e.g., iron oxide, gold) add functionalities such as magnetism or photothermal conversion and present ligands in multivalent patterns. Surface characteristics like size, charge, and coating dramatically influence lymphatic targeting and immune cell uptake.^[^ ^32^ ^]^ **Table** **1** and **Figure** **1** and **2** summarize representative strategies aimed at expanding or enhancing Tregs to promote tolerance, whereas **Table** **2** outlines approaches designed to inhibit/deplete or reprogram Treg function in tumor settings.

**Table 1:** Representative NP strategies to enhance Tregs for tolerance induction.

Strategy	Nanomaterial and cargo	Mechanism	Ref
Antigen‐specific tolerance	Iron‐oxide NP coated with high‐density self‐peptide‐MHC	Act as artificial APC presenting autoantigen; induce antigen‐specific Treg expansion	[35]
Apoptotic tolerogenic signal	PLGA NP loaded with encephalitogenic peptide	Targeted uptake by splenic macrophages (MARCO) → promote Treg activity, induce abortive T cell activation, establish T cell anergy	[36]
Cytokine delivery for Tregs	Anti‐CD4 antibody‐coated PLGA NP encapsulating IL‐2 + TGF‐β	Local paracrine cytokine delivery to CD4^+^ T cells → Treg stabilization/expansion	[40]
	Anti‐CD2/4 antibody‐coated PLGA NP encapsulating IL‐2 + TGF‐β	Local paracrine cytokine delivery to T cells → CD4^+^ and CD8^+^ Treg expansion	[41]
	Anti‐CD2 antibody‐coated PLGA NP encapsulating IL‐2 + TGF‐β	Generate tolerogenic DCs; local paracrine cytokine delivery to CD4^+^ and CD8^+^ T cells → CD4^+^ and CD8^+^ Treg expansion	[84]
	TCR signal‐responsive IL‐2 nanogel attached to ex vivo expanded Tregs	Release IL‐2 upon TCR stimulation, boosting Treg survival/function at antigen site	[85]
	Anti‐CD4/8 antibody‐coated PLGA NP encapsulating IL‐2 + TGF‐β	Local paracrine cytokine delivery to T cells → CD4^+^ and CD8^+^ Treg induction/stabilization	[90]
Epigenetic modulation	Anti‐CD4 nanolipogel delivering 5‐azacytidine	Augment FoxP3 expression in CD4^+^ T cells → expand functional Tregs	[42]
	PLGA NP coencapsulating aVD_3_ and OVA	Create tolerogenic DCs → induce Tregs; Suppress OVA‐specific CTL activity	[47]
Metabolic immunomodulation	PLGA NPs encapsulating PHCCC (mGluR4 agonist)	Controlled PHCCC release is taken up dose‐dependently by DCs → lower CD40/CD80/CD86 and pro‐inflammatory cytokines → skew T cell responses from Th17 toward Tregs	[48]
	PEGylated liposomes encapsulating PHCCC	Suppress DC inflammatory cytokines; curb myelin‐reactive T cell proliferation/IFN‐γ; promote Treg bias	[49]
Cosignaling modulation	PLGA‐Ni microspheres loaded with antisense oligonucleotides against CD40, CD80, and CD86	Create tolerogenic DCs; expand antigen‐specific Tregs	[50]
	PD‐L1‐Fc/Oxi‐αCD NP	Release PD‐L1‐Fc in inflamed gut; amplify Tregs, Th1 and Tfh; suppress Th17	[51]
	LNP carrying mRNA‐PD‐L1	Generate tolerogenic APCs → induce Tregs; reduce activated T cells	[56]
	CLAN PEG‐PLGA NP encapsulating Cas9 mRNA + gRNA against CD40	DC‐targeted CRISPR/Cas9 knockout of CD40 abolishes positive costimulation → skew DCs toward a tolerogenic phenotype → expand Tregs	[86]
Antigen + cosignaling modulation	CLAN PEG‐PLGA NP simultaneously encapsulating autoantigen peptide 2.5 mi, CRISPR‐Cas9 plasmid, and three gRNAs (gCD40, gCD80, gCD86)	DC‐targeted delivery → CRISPR excision of CD40/CD80/CD86 ablates positive costimulation; edited DCs present 2.5 mi on MHC‐II → drive expansion of antigen‐specific Tregs	[55]
Intrinsic immunomodulator NP	Polydopamine NP (also probiotic‐coated)	Inhibit the activation of DCs → elevate Treg/Th17 ratio	[57]
Antigen + NF‐κB inhibitor NP	Egg phosphatidylcholine liposome coloaded with autoantigen and a lipophilic NF‐κB inhibitor (e.g., curcumin)	Suppress APCs’ responsiveness to NF‐κB; induce antigen‐specific Tregs	[58]
Antigen + mTOR inhibitor NP	PLGA/PLA‐PEG NP coloaded with autoantigen and rapamycin	Inhibit the activation of antigen‐specific CD4^+^ and CD8^+^ T cells and B cells; induce antigen‐specific Tregs and Bregs	[59]
		Expand antigen‐specific Tregs	[60]
	LCPs coloaded with Cit‐ME and rapamycin	Generate anti‐inflammatory cytokines and tolerogenic DCs → upregulate Tregs, promote IL‐10 secretion	[61]
	PLGA NP coloaded with HEL_46–61_ peptide and rapamycin	Uptake by BMDCs lowers CD80/CD86 → drives antigen‐specific Treg differentiation	[62]
	PLGA NP coloaded with donor antigen and rapamycin	Modulate indirect T cell activation	[78]
Cytokine + mTOR inhibitor NP	ImmTOR NP (rapamycin) coadministered with Treg‐selective IL‐2 mutein	Synergistically enlarge total and antigen‐specific Tregs; restrain effector‐cell expansion, broaden IL‐2 safety window	[63]
Antigen + AhR agonist NP	AuNP coloaded with AhR agonist ITE and β‐cell autoantigen	Induce tolerogenic DCs → increase Tregs differentiation	[68]
	Nanoliposome coloaded with AhR agonist ITE and myelin oligodendrocyte glycoprotein peptide	Induce tolerogenic DCs → expand antigen‐specific Tregs and Tr1s	[69]
Donor antigen NP	PLGA NP loaded with donor MHC class II peptide	Expand graft‐infiltrating Tregs	[76]
Donor exosomes	Donor‐derived exosomes	Inhibit the Th2 transcription factor, GATA3; induce CD4^+^ T cells to express FoxP3 and TGF‐β	[77]
PI3K/mTOR inhibitor	Chitosan NP encapsulating BEZ235	Suppress effector T cell activation; expand Tregs	[79]
Calcineurin inhibitor	PLGA NP loaded with tacrolimus	Sustain Treg proportions	[83]
LN targeting	PLGA NP coated with MECA‐79 (PNAd‐targeting antibody) and anti‐CD3 mAb	Target NPs to LNs; local anti‐CD3 mAb induces Tregs in graft and draining LNs	[87]
	PLGA NP coated with MECA‐79, carrying anti‐CD40L antibody	Deliver costimulatory blockade specifically to LNs; increases Tregs	[88]
ROS‐scavenging	PEGylated bilirubin NP	Allow the immune system to regain balance → indirectly expand Tregs	[92]

Abbreviations: NP, nanoparticle; MHC, major histocompatibility complex; APC, antigen‐presenting cell; DC, dendritic cell; BMDC, bone‐marrow‐derived dendritic cell; LN, lymph node; PNAd, peripheral node addressin; TCR, T cell receptor; Treg, regulatory T cell; Tr1, type 1 regulatory T cell; Th1/Th2/Th17, T‐helper type 1/2/17 cell; Tfh, T‐follicular‐helper cell; CTL, cytotoxic T lymphocyte; PLGA, poly(lactide‐co‐glycolide); PLA‐PEG, poly(lactic acid)‐poly(ethylene glycol) block copolymer; PEG, poly(ethylene glycol); LNP, lipid nanoparticle; LCP, lipid‐coated calcium‐phosphate nanoparticle; CLAN, cationic‐lipid‐assisted PLGA nanoparticle; MARCO, macrophage receptor with collagenous structure; Ni, nickel; PD‐L1, programmed death‐ligand 1; Fc, fragment crystallizable region; Oxi‐αCD, oxidized α‐cyclodextrin; IL‐2, interleukin‐2; IL‐10, interleukin‐10; IL‐1β, interleukin‐1 beta; IL‐6, interleukin‐6; IL‐12, interleukin‐12; TNF‐α, tumor‐necrosis‐factor alpha; IFN‐γ, interferon gamma; TGF‐β, transforming growth factor β; NF‐κB, nuclear factor κ‐light‐chain‐enhancer of activated B cells; PI3K, phosphoinositide 3‐kinase; mTOR, mechanistic target of rapamycin; ROS, reactive oxygen species; aVD3, 1,25‐dihydroxyvitamin D_3_; OVA, ovalbumin; PHCCC, positive‐allosteric mGluR4 agonist PHCCC; mGluR4, metabotropic glutamate receptor 4; AhR, aryl‐hydrocarbon receptor; ITE, 2‐(1H‐indol‐3‐ylcarbonyl)‐4‐thiazolyl‐ethanamide; Cit‐ME, multiepitope citrullinated peptide; CRISPR, clustered regularly interspaced short palindromic repeats; gRNA, guide RNA; 2.5 mi, insulin‐B:9–23 mimic autoantigen peptide; BEZ235, dual PI3K/mTOR inhibitor; GATA3, GATA‐binding protein 3; mAb, monoclonal antibody.

**Table 2:** Representative NP strategies to inhibit Tregs for antitumor immunity.

Strategy	Nanomaterial and cargo	Mechanism	Ref
Treg targeting	tLyp1‐peptide hybrid NP loaded with imatinib	NRP1‐mediated uptake by intratumoral Tregs; inhibit STAT3 and STAT5 phosphorylation → enhance imatinib effect → downregulate Tregs suppression	[96]
Physical ablation of Tregs	Iron‐oxide NP	NIR‐triggered local hyperthermia selectively kills intratumoral Tregs	[98]
Photo‐inducible tumor ablation + Treg suppression	pH‐responsive layer‐by‐layer NP (PLH/PEG‐PLG) with IR‐780 shell + GITR‐antibody‐decorated PLGA core (imatinib‐loaded)	Tumor acidity destabilizes coatings → corelease of IR‐780 and GITR‐PLGA core; 808 nm NIR activates IR‐780 for synergistic PTT/PDT → immunogenic tumor cell death and DC maturation; GITR‐targeted imatinib blocks STAT3/5–FoxP3 in intratumoral Tregs	[100]
Checkpoint gene silencing	PEG‐PLA NP loaded with siCTLA‐4	Knock down CTLA‐4 in TILs; lower Treg ratio, boost Teff	[101]
	Cationic liposome loaded with siPD‐1 (and doxorubicin)	Reduce Treg infiltration into TME	[102]
TGF‐β‐trap and immune re‐programming	Redox‐sensitive nanomicelle with P17 peptide + PD‐L1 D‐peptide	Neutralize tumor TGF‐β; blocks de‐novo Treg conversion and *FoxP3*.	[103]
Metabolic disruption	GSH‐degradable HMON with siMCT4 + HCPT	Inhibit tumor‐derived lactate; TAMs switch M2 → M1; collapse intratumoral Tregs, expand CD8^+^ T cells	[107]
Treg/Th17 re‐balancing	Bilirubin NP	Modulate gut microbiota → promote Treg/Th17 cell immunity	[108]

Abbreviations: tLyp1, tumor‐penetrating NRP1‐binding peptide; NP, nanoparticle; NRP1, neuropilin‐1; Treg, regulatory T cell; STAT3/5, signal transducer and activator of transcription 3/5; NIR, near‐infrared; PLH, poly(L‐histidine); PEG, poly(ethylene glycol); PLG, poly(L‐glutamic acid); IR‐780, heptamethine cyanine dye IR‐780 iodide; GITR, glucocorticoid‐induced TNF‐receptor‐related protein; PLGA, poly(lactide‐co‐glycolide); PTT, photothermal therapy; PDT, photodynamic therapy; DC, dendritic cell; FoxP3, forkhead box P3; PLA, poly(lactic acid); siCTLA‐4, small‐interfering RNA targeting CTLA‐4; CTLA‐4, cytotoxic T‐lymphocyte‐associated protein 4; TIL, tumor‐infiltrating lymphocyte; Teff, effector T cell; siPD‐1, siRNA targeting PD‐1; PD‐1, programmed cell‐death protein 1; TME, tumor micro‐environment; TGF‐β, transforming growth factor β; P17, TGF‐β‐neutralizing peptide; PD‐L1, programmed death‐ligand 1; GSH, glutathione; HMON, hollow mesoporous organosilica nanoparticle; siMCT4, siRNA targeting monocarboxylate transporter 4; HCPT, hydroxycamptothecin; TAM, tumor‐associated macrophage; M2/M1, anti‐inflammatory/pro‐inflammatory macrophage phenotypes; Th17, T‐helper 17 cell.

### 3.1 Autoimmune and Inflammatory Diseases: Enhancing Treg‐Mediated Tolerance

Autoimmune diseases and chronic inflammatory conditions are characterized by a breakdown of self‐tolerance, in which pathogenic effector T cells attack the body's own tissues. In these settings, augmenting the number or function of Tregs can restore immune balance and ameliorate disease.^[^ ^33^, ^34^ ^]^ Nanomaterials are being harnessed to promote Tregs in two main ways: (a) antigen‐specific tolerogenic NPs and (b) NPs delivering immunomodulators and metabolic agents.

A pioneering approach in nanotherapy for autoimmunity is the use of tolerogenic NPs that act as artificial APCs. These particles are typically coated with disease‐relevant peptide‐MHC complexes or loaded with autoantigens, and they are designed to present antigen to T cells in a noninflammatory context that favors Treg induction. For example, Singha et al. showed that the density of peptide‐MHC (pMHC) on NPs can determine their Treg‐inducing potency. High densities of self‐peptide on iron‐oxide nanocarriers preferentially expanded antigen‐specific Tregs and suppressed autoimmune responses. Mechanistically, densely presented pMHC on NPs drives sustained TCR microcluster formation in cognate T cells in the absence of costimulation, which reprograms antigen‐experienced CD4^+^ T cells toward IL‐10‐producing Tr1‐like regulatory cells and tolerizes effectors, rather than promoting effector differentiation.^[^ ^35^ ^]^ In a different study, inert poly(lacticco‐glycolic acid) (PLGA) NPs loaded with an encephalitogenic peptides were administered intravenously in an experimental autoimmune encephalomyelitis (EAE) model. These particles were avidly taken up by splenic macrophages expressing the scavenger receptor MARCO, which led to long‐term T cell tolerance. This tolerogenic outcome was mediated in part by Treg activity, abortive activation of autoreactive T cells, and the induction of T cell anergy.^[^ ^36^ ^]^

In addition to antigen‐specific signals, nanomaterials are being used to deliver immunomodulators that create a Treg‐favoring environment. A prominent example is the delivery of cytokines like IL‐2 and TGF‐β, a combination known to drive *FoxP3* expression in naïve T cells.^[^ ^37^, ^38^ ^]^ However, using soluble IL‐2 or TGF‐β is challenging due to their pleiotropic effects on T cells, B cells, and NK cells and short half‐lives, which limit the bioavailability to the intended T cell targets.^[^ ^39^ ^]^ To overcome this, McHugh et al. encapsulated IL‐2 and TGF‐β together in biodegradable PLGA NPs and coated the particle surface with anti‐CD4 antibodies. This design enabled the NPs to target CD4^+^ T cells and locally release the cytokines at their surface, acting as a tolerogenic, cytokine‐delivering artificial APCs that localizes Signal 3 (IL‐2/TGF‐β) to T cells during ongoing TCR‐pMHC engagement with endogenous APCs. This cis‐delivery ensures high pericellular cytokine levels that drive the IL‐2R‐JAK1/3‐STAT5 and the TGF‐βR‐SMAD2/3 axis required for FoxP3 induction and stabilization, thereby driving the stabilization and expansion of Tregs.^[^ ^40^ ^]^ Building on this concept, subsequent studies applied dual antibody targeting by coating NPs with both anti‐CD4 and anti‐CD2 to engage a broader range of T cell subsets. Anti‐CD2 (which binds LFA‐2 on T cells) helps target CD8^+^ T cells as well, enabling the induction of both CD4^+^ and CD8^+^ Tregs in vivo. These CD8^+^ Tregs, though less common, can contribute to tolerance. The combination of multitargeted NPs encapsulating IL‐2 plus TGF‐β significantly suppressed murine lupus by expanding the Treg pool in a sustained and antigen‐nonspecific manner.^[^ ^41^ ^]^

Beyond cytokines, an array of other agents has been incorporated into tolerogenic nanocarriers. For instance, liposomal delivery of 5‐azacytidine (5‐azaC), a DNA methyltransferase inhibitor, to CD4^+^ T cells in lupus‐prone mice increased *FoxP3* expression and the number and function of Tregs, resulting in amelioration of lupus nephritis without including lupus autoantigens in the microsphere formulation.^[^ ^42^ ^]^ Mechanistically, 5‐azaC traps DNMTs to induce DNA hypomethylation; durable Treg lineage stability requires demethylation of the FOXP3 CNS2/TSDR enhancer.^[^ ^43^, ^44^ ^]^ The active vitamin D metabolite 1,25‐dihydroxyvitamin D_3_ reprograms dendritic‐cell epigenetic and transcriptional states to enforce a stable tolerogenic phenotype.^[^ ^45^, ^46^ ^]^ When this metabolite was coencapsulated with ovalbumin in PLGA NPs, the treated DCs downregulated MHC‐II, CD80 and CD86, secreted fewer pro‐inflammatory cytokines while increasing IL‐10 and TGF‐β, induced Tregs, and conferred both systemic and oral antigen tolerance in mice.^[^ ^47^ ^]^ Jewell et al. encapsulated the metabotropic glutamate receptor‐4 (mGluR4) agonist PHCCC in PLGA NPs and liposomes to examine whether metabolic reprogramming of immune cells could deepen tolerance. Nanocarrier delivery substantially diminished the systemic toxicity seen with soluble PHCCC yet retained efficient, dose‐dependent uptake by DCs. When these cells were stimulated with lipopolysaccharide, PHCCC‐loaded particles limited the upregulation of CD40, CD80 and CD86, curtailed T cell proliferation, fostered expansion of Tregs and reduced IFN‐γ secretion. In the EAE model, treatment with PHCCC NPs delayed disease onset and alleviated clinical severity relative to the free drug.^[^ ^48^, ^49^ ^]^

Further, NPs that interfere with T cell cosignaling pathways have demonstrated therapeutic potential. For example, biodegradable PLGA‐Ni (nickel ion) microspheres that deliver antisense oligonucleotides (ASOs) against CD40, CD80, and CD86 attenuated autoreactive T cell activation and increased antigen‐specific Tregs, even reversing hyperglycemia in diabetic mice.^[^ ^50^ ^]^ Another example is leveraging NPs to deliver soluble checkpoint ligands that engage inhibitory receptors on T cells or other leukocytes. Reactive oxygen species‐responsive lipid‐polymer hybrid NPs displaying PD‐L1‐Fc were administered orally in a colitis model and programmed to release PD‐L1‐Fc within the oxidatively inflamed gut. Local presentation of PD‐L1 increased Treg frequencies, reduced pro‐inflammatory cytokines, corrected dysbiosis, and markedly attenuated colitis. Mechanistically, PD‐L1 engagement of PD‐1 delivers an inhibitory signal via SHP‐2, dampening TCR‐driven proliferation and cytokine production.^[^ ^51^, ^52^ ^]^ In inflamed settings, PD‐1 signaling biases responses toward regulation by promoting anergy/iTreg conversion of conventional T cells (Tconvs) and by supporting the maintenance and suppressive function of induced Tregs.^[^ ^53^, ^54^ ^]^ Wang et al. also designed a single “all‐in‐one” cationic‐lipid‐assisted PLGA NP (CLAN) that coencapsulated an islet autoantigen peptide (2.5 mi), a CRISPR‐Cas9 plasmid and three guide RNAs targeting CD40, CD80, and CD86. After preferential uptake by DCs, Cas9 ablated expression of the positive costimulatory ligands, converting the edited cells into a tolerogenic state, while concurrent presentation of 2.5 mi drove robust expansion of cognate Tregs. The formulation completely prevented autoimmunity to islet components and inhibited type 1 diabetes development.^[^ ^55^ ^]^ Nanotherapy with mRNA can be used to induce immunoregulatory proteins transiently in vivo. Wang et al. reported that LNPs delivering mRNA encoding PD‐L1 could transiently endow DCs with this checkpoint. In mouse models of rheumatoid arthritis and ulcerative colitis, subcutaneous administration of PD‐L1‐mRNA LNPs induced Tregs and decreased activated T cells, resulting in significant disease amelioration.^[^ ^56^ ^]^

Complementary to ligand‐based targeting, dopamine‐derived polydopamine NPs (PDNI) represent an intrinsically immunomodulatory platform composed solely of polymerized dopamine that gradually releases trace dopamine over time. PDNI suppressed DC maturation, as shown by lower surface expression of CD86 and MHC class II, shifted cytokine production toward an anti‐inflammatory profile by increasing IL‐10 while reducing IL‐1β, IL‐6, and TNF‐α, and elevated the Treg/Th17 ratio both in vitro and in DSS‐ or TNBS‐induced colitis models, thereby attenuating intestinal inflammation. When an ≈30 nm PDNI shell was deposited on probiotic *E. coli* Nissle 1917, the hybrid (EcN@PDNI) combined microbiota restoration with the same local dopaminergic cues, yielding synergistic relief of colitis that surpassed standard aminosalicylic acid therapy.^[^ ^57^ ^]^

Combining antigen‐specific and immunomodulatory signals in the same NP has shown synergistic benefits for inducing tolerance. For instance, liposomes simultaneously delivering arthritogenic antigens and NF‐κB inhibitors (e.g., curcumin, quercetin, or Bay11‐7082) markedly expanded antigen‐specific Tregs and reduced the severity of arthritogenic antigen‐induced inflammatory arthritis, because pharmacologic NF‐κB blockade in the DCs that take up the particles suppressed their pro‐inflammatory response and converted them into a tolerogenic phenotype that preferentially drove Treg differentiation.^[^ ^58^ ^]^

A widely studied approach to increasing Treg number and function is to exploit the greater dependency of effector T cells on mTOR function compared with Tregs. When coadministered with IL‐2, the mTOR inhibitor rapamycin favors the generation, differentiation, survival, and suppressor function of CD4^+^CD25^+^Foxp3^+^ Tregs,^[^ ^59^ ^]^ while at the same time impairing effector T cell proliferation, thereby increasing the Treg/effector T cell ratio and, hence, immunoregulatory activity. Importantly, rapamycin‐loaded PLGA NPs showed significantly stronger immunomodulatory effects than free rapamycin, suggesting that nanoformulation resulted in a higher and more sustained local concentration of rapamycin, increasing the potency of rapamycin as a tolerogenic therapeutic.^[^ ^8^ ^]^ When combined with relevant autoantigens, these NPs profoundly induced antigen‐specific Treg responses in autoimmune models. For example, poly(lactic‐co‐glycolic acid)/poly(lactic acid)‐poly(ethylene glycol) block copolymer (PLGA/PLA‐PEG) NPs encapsulating rapamycin together with myelin peptides efficiently induced and expanded antigen‐specific Tregs, significantly ameliorating EAE.^[^ ^8^, ^60^ ^]^ In a model of rheumatoid arthritis, lipid‐coated calcium phosphate NPs (LCPs) coloaded with multiepitope citrullinated peptides (Cit‐ME) and rapamycin promoted antigen‐specific tolerance by inducing tolerogenic DCs, substantially expanding Treg proportions, elevating anti‐inflammatory IL‐10 levels, and suppressing pathogenic cytokines and autoantibody production, ultimately leading to reduced joint inflammation.^[^ ^61^ ^]^ Extension to cutaneous autoimmunity has been demonstrated with PLGA NPs incorporating rapamycin plus the hen‐egg‐lysozyme autoantigen HEL_46‐61_, which, after uptake by bone‐marrow‐derived DCs (BMDCs), reduce CD80/CD86 expression, induce antigen‐specific Tregs in vitro, expand skin‐homing Tregs in TrpHEL mice, and halt vitiligo progression while shifting the cytokine milieu toward dominant IL‐10.^[^ ^62^ ^]^ Beyond antigen codelivery, rapamycin NPs can also act synergistically with cytokine‐based approaches. ImmTOR NPs coadministered with a Treg‐selective IL‐2 mutein synergistically expanded total and antigen‐specific Tregs, protected against type 1 diabetes and primary biliary cholangitis.^[^ ^63^ ^]^

Innovative combinational strategy has been shown to harness the aryl hydrocarbon receptor (AhR) pathway. Yeste et al. developed stable gold NPs (AuNPs) coloaded with the AhR agonist 2‐(1′H‐indole‐3′‐carbonyl)‐thiazole‐4‐carboxylate (ITE), known to promote Treg generation,^[^ ^64^, ^65^, ^66^, ^67^ ^]^ and the β‐cell autoantigen proinsulin. Upon intravenous administration in nonobese diabetic (NOD) mice, these NPs reprogrammed DCs into a tolerogenic phenotype in vivo, markedly enhancing differentiation of Tregs without requiring ex vivo manipulation. This treatment effectively prevented autoimmune diabetes onset.^[^ ^68^ ^]^ The broader applicability of this AhR‐based approach was confirmed in a multiple sclerosis model: nanoliposomes carrying the AhR agonist ITE along with a myelin oligodendrocyte glycoprotein (MOG_35‐55) peptide significantly expanded antigen‐specific Tregs and mitigated EAE.^[^ ^69^ ^]^

### 3.2 Transplantation: Promoting Tregs for Immune Tolerance

Inducing immune tolerance is a central goal in transplantation, to prevent rejection of allografts in solid organ transplantation and mitigate graft‐versus‐host disease (GvHD) after allogeneic hematopoietic stem cell transplantation (allo‐HSCT).^[^ ^70^, ^71^ ^]^ Tregs are pivotal in these settings: higher Treg frequencies correlate with tolerance in transplant recipients, and adoptive Treg therapy has shown promise in early clinical trials for solid organ transplantation and allo‐HSCT.^[^ ^72^, ^73^, ^74^, ^75^ ^]^ Nanomaterials are being developed to reinforce Treg‐mediated tolerance in transplant settings, often by building on the strategies used in autoimmunity.

In solid organ transplantation, the desired outcome is donor‐specific tolerance, in which Tregs selectively suppress responses to the graft while sparing global immunity. Nanotechnology‐based strategies discussed herein encompass the targeted delivery of antigen‐specific nanovaccines, immunomodulatory drugs, tolerogenic cues, nucleic acids, and focal immunosuppressive agents directly to lymphoid tissues. For example, Shah et al. encapsulated a donor‐derived MHC class II peptide into PLGA NPs and administered them to mice receiving an MHC‐mismatched skin transplant. The peptide‐NP treatment resulted in significantly prolonged graft survival. Mechanistically, host APCs internalize the peptide‐NPs and present the donor peptide, but in the absence of inflammatory adjuvant signals, these APCs remain semi‐immature. Such a state tends to drive T cells toward FoxP3^+^ regulatory differentiation or deletion, especially for high‐avidity T cells recognizing the donor peptide.^[^ ^76^ ^]^ Not all nanocarriers in this context are synthetic. Exosomes and other extracellular vesicles (60–100 nm in size) can function as natural NPs. Song et al. showed that donor‐derived exosomes carry MMP1α, which induced the donor antigen‐specific Tregs, attenuated the T helper (Th)2‐skewed inflammation in cardiac allografts, and promoted the graft survival.^[^ ^77^ ^]^

Nanocarriers have also synergized with systemic immunosuppressants to induce tolerance. Luo et al. formulated PLGA NPs conjugated with donor antigens for administration to fully MHC‐mismatched mice receiving islet cell transplants. When this nanotherapy was paired with a short course of low‐dose rapamycin, long‐term graft acceptance was achieved in ≈60% of transplant recipients (versus ≈20% with the NPs alone). Although the underlying mechanism was not fully investigated, it is plausible that rapamycin expanded peripheral Tregs, thereby reinforcing the tolerogenic milieu initiated by the donor‐antigen NPs.^[^ ^78^ ^]^ In a separate study, Xing et al. encapsulated BEZ235, a dual PI3K/mTOR inhibitor, into chitosan NPs to test in a mouse heart transplant model. This nanoformulation preferentially accumulated in lymphoid organs, reduced CD4^+^ and CD8^+^ effector T cells, and increased Treg frequency, which correlated with attenuated acute rejection.^[^ ^79^ ^]^ Nanomaterials can also improve the delivery of conventional immunosuppressive drugs and increase their tolerogenic capacity. Tacrolimus (FK506), for instance, is a calcineurin inhibitor commonly used to prevent organ rejection, but high systemic exposure can be nephrotoxic and may even impair Tregs.^[^ ^80^, ^81^, ^82^ ^]^ Cao et al. formulated FK506‐loaded PLGA NPs for testing in vascularized composite allografts. The slow‐release profile of these NPs enabled effective immunosuppression at markedly lower peak drug levels. Notably, animals treated with FK506‐NPs maintained elevated Treg proportions for a longer period, and the transplanted grafts survived significantly longer than in animals given free drug, in which Treg levels declined and rejection ensued.^[^ ^83^ ^]^

An alternative strategy is to deliver tolerogenic cytokine signals directly to promote Treg differentiation in situ. Horwitz et al. recently developed biodegradable PLGA NPs that were coated with anti‐CD2 antibodies to target T cells and encapsulated IL‐2 and TGF‐β. These NPs effectively functioned as “artificial APCs” by engaging T cells through CD2 while simultaneously delivering IL‐2/TGF‐β signals to drive *FoxP3* expression. In a mouse model, treatment with these cytokine‐loaded NPs expanded both CD4^+^ and CD8^+^ Tregs by 4–5‐fold and prolonged allograft survival.^[^ ^84^ ^]^ In parallel to these in vivo approaches, nanotechnology has been applied ex vivo to enhance adoptive Treg cell therapies. Eskandari et al. engineered Tregs with a TCR signal‐responsive IL‐2 nanogel “backpack”. These cytokine nanogels remained attached to the Treg surface and released IL‐2 whenever the local redox potential rises, as occurred at the membrane when the TCR was engaged. Thus, the transferred Tregs carried an on‐board IL‐2 supply that was unleashed precisely at sites of antigen encounter, boosting their survival and suppressive potency. In murine skin transplant models, Tregs equipped with IL‐2/Fc nanogels suppressed alloimmune responses more effectively and prolonged allograft survival with conventional Tregs lacking nanogels.^[^ ^85^ ^]^

Within the portfolio of nucleic‐acid nanotherapies, Wang et al. engineered PEG‐PLGA CLAN that coencapsulated Cas9 messenger RNA together with a single‐guide RNA directed against CD40. These CLAN‐mCas9/gCD40 particles efficiently transfected DCs, excised the CD40 locus, and markedly reduced surface CD40 expression. This shift favored the expansion of Tregs. When administered intravenously in an acute murine skin allograft model, the edited DC phenotype curtailed T cell activation, limited graft damage, and substantially extended graft survival, thereby demonstrating a practical route to transplant tolerance through in situ CRISPR editing of costimulatory pathways.^[^ ^86^ ^]^

Another emerging tactic is to focus immunosuppressive agents specifically to lymphoid tissues, thereby enhancing their tolerogenic effects at the primary sites of immune activation. High endothelial venules in lymph nodes uniquely express peripheral node addressin (PNAd), which can be targeted by the MECA‐79 antibody. Bahmani et al. took advantage of this feature by coating NPs with MECA‐79 and loading them with an anti‐CD3 monoclonal antibody (mAb). The goal was to deliver a subclinical “stimulus” to T cells specifically in lymph nodes to induce tolerance. Short‐term treatment of cardiac allograft recipients with these lymph nodes (LN)‐homing anti‐CD3 NPs significantly prolonged allograft survival, accompanied by a marked increase in intragraft and draining‐LN Tregs. Notably, if Tregs were experimentally depleted, the graft‐prolonging effect of the NP therapy was abrogated, indicating that tolerance was mediated through a Treg‐dependent mechanism.^[^ ^87^ ^]^ Similarly, Zhao et al. developed MECA‐79‐decorated NPs carrying anti‐CD40L. In a mouse heart transplant model, these LN‐targeted NPs markedly delayed rejection and increased the local Treg proportion relative to nontargeted therapy; when paired with low‐dose rapamycin, they achieved far longer graft survival than soluble anti‐CD40L plus rapamycin.^[^ ^88^ ^]^ By concentrating immunomodulatory drugs in lymphoid organs, such nanocarriers amplify tolerogenic signals where Tregs are induced, while limiting broad systemic immunosuppression.

In allo‐HSCT, a main immune hurdle is GvHD, in which donor effector T cells recognize host tissues as foreign and attack organs of the recipient. Effective GvHD prophylaxis can be achieved in settings where sufficient numbers of functional Tregs are present at sites of GvHD initiation, amplification, and tissue damage.^[^ ^71^, ^89^ ^]^ Interestingly, in human‐antimouse xenogeneic GvHD model, the cytokine‐loaded NP approach produced qualitatively distinct Treg populations. For example, Tregs induced in vivo by IL‐2/TGF‐β NPs had higher expression of stability‐associated markers, including CD45RA on CD4^+^ Tregs and CD122 on CD8^+^ Tregs, than Tregs induced by administering the same cytokines in soluble form.^[^ ^90^ ^]^ ImmTOR NPs coadministered with a high‐affinity IL‐2 mutein further broadened the therapeutic window of engineered IL‐2 by preferentially expanding Tregs, restricting effector T cell proliferation, and preventing disease exacerbation in murine GvHD models.^[^ ^63^ ^]^

An alternative strategy is to utilize nanomaterials to dampen the early inflammatory triggers of GvHD, thereby providing a permissive environment to facilitate the generation and reconstitution of Tregs that blunt GvHD. Bilirubin is a natural anti‐inflammatory and antioxidant; Lee et al. developed PEGylated bilirubin NPs (BRNP) that can scavenge reactive oxygen species and inhibit inflammatory cytokine release.^[^ ^91^ ^]^ In a murine major MHC‐mismatched bone marrow transplant model, administration of these BRNPs during the peri‐transplant period reduced tissue damage caused by excessive cytokine production, referred to as a “cytokine storm,” and/or alloreactive donor effector T cells that amplify tissue injury. Although BRNPs did not directly expand Tregs, the immune system regained balance, promoting immune tolerance through the preservation of Treg function.^[^ ^92^ ^]^ This approach complements Treg‐directed strategies. The optimal scenario for transplant tolerance after allo‐HSCT may be a dual approach in which inflammation is controlled with agents such as BRNPs or other nanoformulations, while Tregs are expanded using cytokine‐ or antigen‐loaded NPs, to establish durable immune tolerance.

### 3.3 Tumor Immunotherapy: Targeting and Reprogramming Tregs

In the tumor microenvironment (TME), Tregs undergo metabolic adaptation that sustains suppression of cytotoxic T cell function and limits the efficacy of antitumor immunotherapy.^[^ ^93^, ^94^ ^]^ Conventional systemic depletion of Tregs—such as via anti‐CD25 antibodies—can provoke broad immune activation and risk autoimmunity.^[^ ^95^ ^]^ In contrast, nanotechnology offers localized and cell‐specific strategies to weaken intratumoral Tregs while preserving overall immune balance and homeostasis.

To reinvigorate antitumor immune responses, Tregs in the TME can be directly targeted for depletion or functional inhibition. In one such illustration, Ou et al. constructed tLyp1 peptide‐conjugated hybrid NPs that specifically target Neuropilin‐1 (NRP1), a surface receptor highly expressed on Tregs, and used them to deliver a tyrosine kinase inhibitor, imatinib, into melanoma in vivo. Imatinib has a notable immune effect: it can limit Treg accumulation by suppressing IDO expression in tumor‐infiltrating cells.^[^ ^96^, ^97^ ^]^ Another approach is NP‐mediated thermal ablation of intratumoral Tregs. Chen et al. demonstrated that systemically delivered iron‐oxide NPs can act as photothermal agents that preferentially accumulate in tumor‐associated Tregs. After near‐infrared laser irradiation of the tumor, the NPs converted light to heat, producing localized hyperthermia that selectively ablated intratumoral Tregs. This NP‐enabled photothermal therapy led to marked Treg depletion within tumors and, when combined with an immune checkpoint inhibitor, elicited robust antitumor T cell responses and tumor regression.^[^ ^98^, ^99^ ^]^ Bridging these two strategies, a pH‐responsive, layer‐by‐layer hybrid nanoplatform has been described that coreleased the photothermal/photodynamic dye IR‐780 and imatinib‐loaded, GITR‐targeted PLGA cores. Light activation provoked immunogenic tumor cell death and DC priming, which in conjunction with imatinib concomitantly diminished Treg suppressive activity, thereby synergistically amplifying CD8^+^‐mediated tumor clearance.^[^ ^100^ ^]^

NP strategies have been used to reprogram intratumoral Tregs rather than eliminate them outright. Blockade of Treg immunoinhibitory receptors or intracellular signals that support Tregs and their function. For instance, Mi et al. formulated PEG‐PLA polymer NPs carrying siRNA against CTLA‐4, aiming to knock down CTLA‐4 in T cells (including Tregs). In a melanoma model, systemic administration of these NP‐siCTLA‐4 particles reduced CTLA‐4 expression and increased the ratio of effector T cells to Tregs in the TME, thereby enhancing antitumor immunity without significantly expanding Treg numbers.^[^ ^101^ ^]^ Similarly, liposomal NPs loaded with siRNA against PD‐1 have been used to transiently silence PD‐1 expression on T cells in tumor‐bearing mice. This intervention lowered PD‐1 levels on intratumoral T cells and reduced the proportion of Tregs in the tumor. When combined with low‐dose liposomal doxorubicin, Tregs were further depleted, tumor growth was delayed, and median survival was extended.^[^ ^102^ ^]^

Another mechanism involves neutralizing Treg‐supporting soluble factors such as TGF‐β in the TME. Zhao et al. developed a “nanoextinguisher,” a reduction‐sensitive lipid/polymer micelle decorated with a fibroblast‐activation‐protein‐cleavable linker and loaded with both a TGF‐β trap peptide (P17) and a PD‐L1‐blocking peptide. After accumulating in the tumor, these NPs released the TGF‐β trap in response to the tumor protease environment, thereby neutralizing tumor‐derived TGF‐β and preventing the recruitment or differentiation of new Tregs. In an orthotopic 4T1 breast cancer model, this strategy impaired the influx of new Tregs and curbed *FoxP3* expression in the TME, functionally inactivating the remaining Tregs. The resulting relief of Treg‐mediated immunosuppression allowed local DCs and CD8^+^ T cells to mount effective antitumor responses, especially when paired with photodynamic therapy or a cancer vaccine.^[^ ^103^ ^]^

Innovative nanocarriers have also been designed to target metabolic dependencies of Tregs within tumors. Tumor cells frequently rely on aerobic glycolysis, producing and exporting lactate via monocarboxylate transporters (MCTs). Lactate accumulation leads to local acidification (lower pH), which impairs cytotoxic T cell function and polarizes macrophages towards an M2 phenotype.^[^ ^104^, ^105^ ^]^ Conversely, Tregs have been shown to utilize lactic acid as a fuel via MCT1, and lactic acid can enhance FoxP3 expression under some circumstances.^[^ ^106^ ^]^ To exploit this, one group designed a glutathione (antioxidant)‐degradable hollow mesoporous organosilica nanocapsule (HMON) coloaded with hydroxycamptothecin and an siRNA targeting MCT‐4, a MCT required for lactate export from tumor cells. In B16F10 melanoma and 4T1 tumor models, systemic HMON delivery caused a sharp drop in extracellular lactate levels in the tumor. This metabolic disruption repolarized tumor‐associated macrophages from an anti‐inflammatory M2 to a pro‐inflammatory M1 phenotype, reduced Tregs, and restored the activity of CD8^+^ T cells.^[^ ^107^ ^]^ Extending this metabolic strategy, systemic administration of the antioxidant BRNP, described above, rewired the tumor‐gut axis by reshaping the microbiota and their metabolism to promote a favorable Treg/Th17 balance, which in turn restrained Lewis lung‐carcinoma growth.^[^ ^108^ ^]^

## 4 Tregs in Immune Responses to Nanomaterials: Unintended Immunological Consequences

Nanomaterials, especially when administered in vivo, interact with the immune system in complex ways. In some cases, the body mounts an inflammatory response to the NP as a foreign substance; in other cases, NPs can evoke anti‐inflammatory or regulatory responses that involve Tregs or Treg‐associated cytokines. This section discusses how Tregs come into play during immune responses to NPs, focusing on two scenarios: (a) NPs inadvertently inducing immunosuppressive, Treg‐favoring conditions, and (b) Tregs limiting the efficacy of NP‐based immunotherapies or vaccines.

### 4.1 NP Exposure Eliciting Treg Responses

While many NP formulations are designed to be immunologically “stealthy” (e.g., PEGylated surfaces to avoid detection),^[^ ^109^ ^]^ several studies have shown that certain NPs can actively skew the immune system toward a suppressed or tolerogenic state.^[^ ^110^, ^111^ ^]^ An illustrative case is that of inert polystyrene NPs. Mohamud et al. demonstrated that an inert 50 nm polystyrene NP (coated with glycine to reduce toxicity) can act as an immune “imprinting” agent in the lung, biasing the local immune environment toward tolerance. Mice pre‐exposed to these polystyrene NPs became resistant to developing allergic inflammation in a subsequent asthma challenge. Mechanistically, the NP exposure expanded the population of Tregs in the lungs, specifically increasing the proportion of highly suppressive TNFR2^+^ Foxp3^+^ Tregs. The NP treatment also promoted CD103^+^ tolerogenic DCs in the lung, which are known to induce Foxp3^+^ Tregs and maintain mucosal tolerance. These findings provide direct evidence that engineered NPs alone can promote selective expansion of maximally suppressive Tregs in vivo.^[^ ^112^ ^]^ In addition, fullerene crystalline C_60 (nano‐C_60) was reported to attenuate a T cell‐mediated hypersensitivity reaction in mice. Nano‐C_60 treatment shifted the cytokine profile toward a Th1‐dominant and anti‐inflammatory pattern: it suppressed IL‐6 and IL‐17 while increasing TNF‐α, and notably it increased the ratio of Tregs among CD4^+^ T cells. The authors concluded that the mitigation of inflammation by fullerenes was likely due to an elevated Treg response and inhibition of Th17.^[^ ^113^ ^]^ Mitchell et al. showed that inhalation of multiwalled carbon nanotubes (MWCNTs) in mice suppressed the antibody response to an unrelated antigen delivered later.^[^ ^114^ ^]^ This remote effect was traced to alveolar macrophages in the lung that released TGF‐β after nanotube exposure; the TGF‐β entered circulation and induced a cascade resulting in elevated IL‐10 production in the spleen, thereby dampening systemic immune reactivity. Although the study did not directly measure Treg frequencies, it is plausible that Tregs were mobilized or functionally enhanced as part of this generalized suppressive response given the known role of TGF‐β and IL‐10 in Treg biology. Supporting this notion, Blank et al. found that poly(vinyl alcohol)‐coated superparamagnetic iron oxide NPs (PVA‐SPIONs) altered DC function in a manner that favored Tregs. These NPs, at noncytotoxic doses, did not prevent DCs from taking up antigen, but they interfered with antigen processing and DC activation. Upon stimulation, treated DCs showed a sharp reduction in pro‐inflammatory cytokines (e.g., IL‐1β, IL‐6, IL‐12, and TNF‐α) and, notably, an enhancement of IL‐10 production and an associated decrease in T cell proliferation.^[^ ^115^ ^]^ This phenotype, characterized by impaired antigen presentation together with high IL‐10, is consistent with a tolerogenic DC population that can drive Treg responses.^[^ ^116^ ^]^

In addition to IL‐10 and TGF‐β, NPs can induce other changes consistent with a tolerogenic milieu. AgNPs, for instance, have well‐documented anti‐inflammatory properties: exposure of macrophages to AgNPs can downregulate proinflammatory mediators like TNF‐α and IL‐6.^[^ ^117^ ^]^ In vivo, topical application of AgNPs to wounds was shown to increase TGF‐β1 while reducing IL‐6 at the wound site, fostering a microenvironment conducive to healing and immune quiescence.^[^ ^118^ ^]^ AuNPs, when appropriately coated, have also exhibited immunosuppressive potential. For example, one study reported that small citrate‐coated AuNPs (5 nm) disrupted IL‐1β‐mediated inflammatory signaling in immune cells.^[^ ^119^ ^]^ Similarly, iron oxide NPs (IONPs) used as imaging agents have demonstrated immunosuppressive properties; in mice, a single dose of carboxydextran‐coated IONPs (Ferucarbotran, “Resovist”) administered 1 h before ovalbumin immunization markedly suppressed the subsequent immune response, as shown by reduced OVA‐specific IgG_1_/IgG_2_a titers and diminished IFN‐γ and IL‐4 secretion from splenocytes upon antigen restimulation. This indicates that IONP exposure compromised both humoral and Th1/Th2 cellular immunity to the antigen.^[^ ^120^ ^]^

Thus, these data suggest certain NPs can skew innate immune cells toward a regulatory, Treg‐permissive state. Collectively, these observations underscore the concern that engineered nanomaterials might induce inadvertent immune tolerance. From a safety perspective, such unintended immunosuppression might weaken host resistance to infections or tumors if NPs are widely used without attention to their immunological effects.^[^ ^110^ ^]^ On the flip side, if appropriately leveraged, these findings suggest that some NPs possess an intrinsic capacity to dampen overactive immune responses. Indeed, carbon nanotubes have been explored as tools to reduce autoreactive T cell and B cell activity.^[^ ^121^ ^]^ Researchers must carefully evaluate new nanomaterials for these immune‐modulating side effects, distinguishing between detrimental and therapeutically beneficial settings.

### 4.2 Tregs Limiting the Efficacy of NP Therapies

While some nanomaterials unintentionally promote Treg‐mediated tolerance, in other cases Tregs themselves become an undesired barrier that undermines the intended efficacy of nanotechnology‐based treatments. This effect is particularly evident in cancer nanomedicine. Many NP therapeutics and vaccines are designed to stimulate the immune system against tumors, such as formulations that deliver tumor‐associated antigens and adjuvants to DCs to provoke a cytotoxic T cell response. In principle, such NP‐based cancer vaccines can generate robust antitumor effector T cell activity.^[^ ^122^, ^123^, ^124^ ^]^ However, the immunosuppressive TME frequently counteracts this activation by expanding suppressor cell populations, most prominently Tregs. Tumors secrete immunomodulatory factors such as TGF‐β, IL‐10, and CCL22, and create conditions like chronic antigen exposure and hypoxia that promote Treg accumulation and activation. These Tregs then become a major barrier to effective therapy by dampening cytotoxic T cell function and reinforcing tolerance to tumor antigens.^[^ ^125^ ^]^ In the context of NP vaccines or immunotherapies, this results in a push‐and‐pull dynamic: the NPs provide stimulatory cues to activate antitumor T cells, while the tumor simultaneously expands Tregs and myeloid‐derived suppressor cells, which impose a tolerogenic program on APCs and directly inhibit effector T cells. The outcome is often a weakened overall immune response despite the use of advanced vaccine strategies. In essence, the host's regulatory feedback mechanisms, exploited by the tumor, sabotage the therapeutic goals of NP‐based interventions.^[^ ^9^ ^]^

Multiple preclinical studies have shown that Tregs markedly limit the efficacy of NP‐based immunotherapies.^[^ ^96^, ^98^, ^103^ ^]^ For example, in a recent vaccine study using NP‐packaged virus‐like particles, the antitumor T cell response was suppressed by Tregs but was markedly enhanced once Tregs were removed. In that model, a CpG‐adjuvanted NP vaccine targeting a tumor peptide expanded both effector T cells and Tregs. When the vaccine was combined with anti‐CD25 antibodies to deplete Tregs, tumor‐bearing mice exhibited significantly improved outcomes, including greater CD8^+^ T cell infiltration into tumors and substantially reduced tumor volumes compared with vaccination alone. Imaging and histological analyses further confirmed that in the absence of Treg depletion, many activated CD8^+^ T cells remained excluded or functionally impaired within the tumor, whereas Treg removal allowed NP‐induced T cells to penetrate the tumor and attack malignant cells effectively.^[^ ^126^ ^]^ These findings align with the broader cancer immunology consensus that Tregs are a critical impediment to successful immunotherapy, including NP‐based therapies.

## 5 Challenges and Outlook

**Figure 3:**
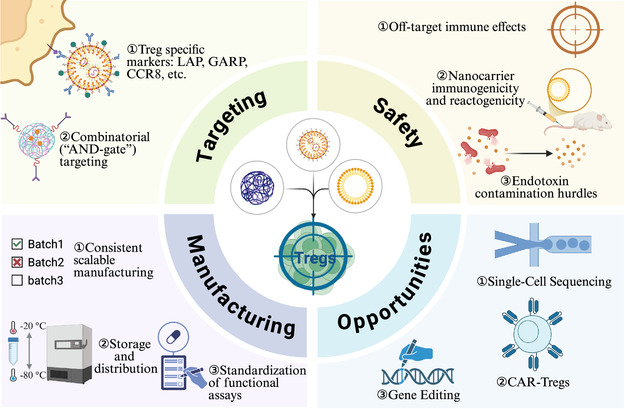
Key challenges and future opportunities at the intersection of nanotechnology and Treg biology. Created in BioRender. Chen, Y. (2025) https://BioRender.com/k0v7nkn.

The intersection of nanotechnology and Treg biology holds substantial promise for next‐generation immunotherapies. We summarize current clinical progress to date in **Table** **3**, noting that most programs remain in phase 1 or phase 2. Translation into practice will require advances in targeting efficiency and specificity, safety and immune toxicity, and manufacturing and standardization, along with future opportunities. The following sections, together with **Figure** **3**, examine these challenges and outline priorities for future work.

**Table 3:** Novel nanomaterial‐based therapies for Treg modulation.

Candidate	Registration number	Status (09/2025)	Trial phase	Nanoplatform/cargo	Indication (population)	Putative Treg‐modulating mechanism
nal‐IRI	NCT01494506	Completed	Phase 3	PEGylated liposomal irinotecan	Metastatic pancreatic cancer	Deplete Treg cells, upregulates MHC‐I and PD‐L1
Nanocurcumin	NCT03140657	Completed	Phase 2	Nanocurcumin capsules (the formulation of curcumin NPs, Exirnanosina)	Ankylosing spondylitis	Balance Treg/Th17 ratio
Nanocurcumin	NCT06281353	Completed	Phase 2, phase 3	Nanocurcumin capsules	Anogenital warts	Decrease the levels of IFN‐γ, FOXP3^+^Treg, and NFκB in lesions
SEL‐212	NCT04513366	Completed	Phase 3	PLGA NPs with sirolimus (ImmTOR) coadministered with pegylated uricase	Chronic refractory gout	ImmTOR induces antigen‐specific tolerance (Treg‐mediated) to coadministered biologic, reducing ADAs
mRNA‐6231	NCT04916431	Completed	Phase 1, Dose‐Escalation	LNP‐delivered mRNA encoding a modified human interleukin 2 mutein fused to human serum albumin (HSA‐IL2m)	Healthy adults	Preferential activation/expansion of Tregs by IL‐2 mutein
TAK‐101	NCT04530123	Active, not recruiting	Phase 2 dose‐ranging	PLGA NPs encapsulating gliadin peptides	Celiac disease	Antigen‐specific tolerance via tolerogenic APC uptake and activation of FOXP3^+^ iTregs/Tr1 cells
CNP‐201	NCT05250856	Terminated (lagging enrollment and administrative reasons)	Phase 1b/2a	PLGA NPs encapsulating purified peanut extract drug substance	Peanut allergy	Pathogenic T cells then undergo deletion and anergy and activate a Treg response through this endogenous mechanism for maintaining peripheral tolerance
CNP‐104	NCT05104853	Active, not recruiting	Phase 2a	PLGA NPs encapsulating PDC‐E2 antigen	Primary biliary cholangitis	
CNP‐106	NCT06106672	Recruiting	Phase 1b/2a	PLGA NPs encapsulating AChR peptides	Generalized myasthenia gravis	
CNP‐103	NCT06783309	Recruiting	Phase 1b/2a	PLGA NPs encapsulating multiple β‐cell antigens	Recent‐onset type 1 diabetes (stage 3)	
PVT201	NCT06798454	Completed	Phase 1	pMHC‐decorated NPs	Primary biliary cholangitis	Convert pathogenic T cells into disease‐regulating Tregs

Abbreviations: NP, nanoparticle; NCT, National Clinical Trial identifier (ClinicalTrials.gov); nal‐IRI, pegylated liposomal irinotecan (nanoliposomal irinotecan); PEG, polyethylene glycol; PEGylated, surface‐modified with polyethylene glycol; PLGA, poly(lactide‐co‐glycolide); LNP, lipid nanoparticle; HSA, human serum albumin; IL‐2, interleukin‐2; IL‐2 m, interleukin‐2 mutein; HSA‐IL2m, human serum albumin–interleukin‐2 mutein fusion; APC, antigen‐presenting cell; Treg, regulatory T cell; iTreg, induced regulatory T cell; Tr1, type 1 regulatory T cell; Th17, T‐helper type 17 cell; FOXP3, forkhead box P3; MHC‐I, major histocompatibility complex class I; PD‐L1, programmed death‐ligand 1; ADA(s), antidrug antibody(ies); PDC‐E2, pyruvate dehydrogenase complex E2 subunit; AChR, acetylcholine receptor; pMHC, peptide‐major histocompatibility complex; PBC, primary biliary cholangitis; NF‐κB, nuclear factor κ‐light‐chain‐enhancer of activated B cells; IFN‐γ, interferon‐γ.

### 5.1 Targeting Efficiency and Specificity

Achieving efficient and specific delivery of nanotherapeutics to Tregs remains a significant challenge. Tregs, unlike phagocytes, do not inherently take up NPs. As a result, systemically administered NPs are often cleared by liver and splenic macrophages before they ever reach Tregs. This makes it hard to achieve a high intracellular payload or functional effect in Tregs in vivo.^[^ ^127^ ^]^ Approaches to improve targeting include decorating NPs with antibodies or ligands that recognize Treg surface markers, thereby promoting selective binding and uptake by Tregs. Early proof‐of‐concept studies support this strategy. For example, one group engineered polyethylene glycol‐coated single‐walled carbon nanotubes (PEG‐SWCNTs) conjugated with antibodies against glucocorticoid‐induced tumor necrosis factor receptor family‐related protein (GITR). GITR is constitutively expressed on Tregs at high levels, is upregulated on activated effector T cells, and is present at intermediate levels on NK cells. These GITR‐targeted nanocomposites showed enhanced binding to Tregs and increased internalization into Tregs via receptor‐mediated endocytosis, demonstrating that receptor‐mediated targeting can markedly increase NP uptake by Tregs.^[^ ^128^ ^]^ Similarly, administration of NPs conjugated with tLyp1, a tumor‐homing and ‐penetrating peptide that specifically binds neuropilin‐1 (NRP1) expressed at high levels on tumor‐associated Tregs, enhanced Treg suppressor function and stability, particularly within the TME, and underscored the feasibility of directing nanocarriers to Tregs in vivo.^[^ ^96^ ^]^ However, translating these approaches to humans necessitates careful target selection: numerous surface markers upregulated on Tregs (e.g., CTLA‐4, GITR, OX40, and CD25) are also expressed on Tconvs, as summarized in **Table** **4**, thereby raising concerns regarding specificity.^[^ ^26^, ^31^, ^129^, ^130^, ^131^, ^132^, ^133^, ^134^, ^135^, ^136^, ^137^, ^138^, ^139^, ^140^, ^141^, ^142^, ^143^, ^144^, ^145^, ^146^, ^147^, ^148^, ^149^, ^150^, ^151^, ^152^, ^153^, ^154^ ^]^ Whereas NRP1 is a promising Treg marker in mice, its expression on human Tregs is less ubiquitous and overlaps with other cell types.^[^ ^155^ ^]^ Recent studies have sought more human Treg‐specific markers. Notably, activated human Tregs uniquely coexpress the latency‐associated peptide (LAP) and glycoprotein A repetitions predominant (GARP), which together anchor latent TGF‐β on the Treg surface.^[^ ^156^, ^157^ ^]^ Tregs that are LAP^+^GARP^+^FoxP3^+^ represent a highly suppressive subset; for example, isolation of LAP^+^ Tregs after ex vivo expansion yields greater than 90% pure FoxP3^+^ Tregs with potent suppressor function.^[^ ^158^ ^]^ Additionally, CD127 expression levels can differentiate human Tconvs from Tregs, being low or absent on resting Tregs and high on most Tconvs.^[^ ^129^, ^159^ ^]^ In the tumor setting, CCR8 is highly enriched on human tumor‐infiltrating Tregs and is already being exploited therapeutically.^[^ ^160^, ^161^ ^]^ Additional candidates include GPA33, which enriches for stable, nonproinflammatory tTregs, and FCRL3, a human‐restricted receptor that marks a subset of Tregs with attenuated responsiveness to exogenous IL‐2.^[^ ^162^ ^]^ These markers are being leveraged to enable selective targeting or enrichment of therapeutic Tregs.

**Table 4:** Markers expressed in both Tregs and conventional T cells.

Marker	Compartment	Typical pattern in Tregs	Typical pattern in Tconv	Refs
CD25 (IL2RA)	Membrane	High/constitutive on Tregs	Inducible on activated Tconv	[129, 130]
CTLA‐4 (CD152)	Membrane (predominantly intracellular pools)	High/constitutive on Tregs	Upregulated after activation on Tconv	[131, 132]
TIGIT	Membrane	High/enriched on Tregs	inducible on activated effector T cells	[133, 134]
LAG‐3 (CD223)	Membrane	Contribute to Treg suppressor activity	Naive T cells express low levels of LAG‐3, and expression increases upon antigen stimulation	[26, 135]
PD‐1 (PDCD1)	Membrane	High on Tregs with an effector phenotype	Expressed on Tonv upon TCR stimulation	[136, 137]
ICOS (CD278)	Membrane	Upregulated after in vivo sensitization on Tregs; promote Tregs survival	High on CD4^+^ effector T cells with high inflammatory potential	[138, 139, 140]
GITR (TNFRSF18/CD357)	Membrane	High on Tregs independent of location and activation state	Upregulated on activated Tconv	[141, 142]
OX40 (TNFRSF4/CD134)	Membrane	High on Tregs; controls T reg‐mediated suppression	Promote Tconv division and survival; augment the clonal expansion of effector and memory populations	[143, 144]
4‐1BB (TNFRSF9/CD137)	Membrane	Treg activation signature	Surrogate marker for antigen‐specific activation of human CD8^+^ T cells	[145, 146]
CD39 (ENTPD1)	Membrane	High on suppressive Tregs; can be induced on activated Tconv subsets	Inducible on activated Tconv	[31, 147, 148, 149]
CD73 (NT5E)	Membrane	High on suppressive Tregs	Upregulated after activation on Tconv	[150]
NRP1 (NRP1)	Membrane	Specific marker of murine Treg; not a reliable human Treg marker	Inducible on activated Tconv	[155]
CCR4 (CD194)	Membrane	High on Tregs	Expressed by Th2 and Th17	[151]
Helios (IKZF2)	Nuclear	Enriched in Tregs and inducible upon activation	Induced during Tconv activation and proliferation	[152]
FOXP3	Nuclear	Specific marker of murine Treg; high/constitutive on human Tregs	Transiently induced on activated human Tconv	[153, 154]

To further enhance specificity, combinatorial (“AND‐gate”) targeting, in which NPs are functionalized with multiple ligands so that only cells coexpressing two or more markers achieve high‐affinity binding and uptake, offers a rational path to discriminate Tregs from activated effector T cells.^[^ ^163^ ^]^ Overall, improving the precision of NP delivery to human Tregs versus other cells remains an active area of research, and success in this area will greatly amplify the therapeutic index of Treg‐modulating nanomedicines.

### 5.2 Safety and Immune Toxicity

Safety is a paramount concern for any new therapy, and nanomedicines that modulate the immune system must be scrutinized for both traditional toxicology and immunological side effects. One set of concerns revolves around off‐target immune effects. For therapies aimed at expanding Tregs, there is a risk of inducing generalized immunosuppression. Patients might become more susceptible to infections or tumors if the treatment drives an excessive Treg response or affects other regulatory pathways. Conversely, strategies that deplete or inhibit Tregs (for cancer) carry a risk of precipitating autoimmunity or inflammatory damage in healthy tissues if they are not sufficiently localized. An example is the use of systemic high‐dose IL‐2 to boost Tregs in autoimmune disease: while IL‐2 can expand Tregs, effector T cells and NK cells can also be activated, leading to paradoxical inflammation or capillary leak syndrome at high doses.^[^ ^164^ ^]^ NP delivery might mitigate some of these issues by improving localization and dose control, but extensive in vivo testing is needed to find a safe therapeutic window.

Another key concern is the immunogenicity and reactogenicity of the NP itself. Many NP formulations (liposomes, polymeric NPs, etc.) consist of materials that can trigger immune recognition. For instance, the PEG polymers commonly used to cloak NPs can themselves provoke anti‐PEG antibody responses in some individuals, leading to accelerated blood clearance of the NP or to allergic reactions upon repeated dosing.^[^ ^165^ ^]^ Complement activation is another issue: certain surface chemistries (particularly cationic or hydrophobic surfaces) can activate the complement cascade, causing infusion reactions known as CARPA (Complement Activation‐Related PseudoAllergy).^[^ ^166^ ^]^ In mice, pre‐exposure to amorphous silica NPs (SiO_2_ NPs) before *Pseudomonas aeruginosa* pneumonia increased mortality without increasing bacterial burden, implicating dysfunction of the alveolar‐capillary barrier rather than enhanced pathogen growth.^[^ ^167^ ^]^ Likewise, pre‐exposure to SWCNTs reduced pulmonary clearance of Listeria monocytogenes and dampened macrophage phagocytosis and nitric‐oxide production over days 3–7 post‐infection, which is consistent with a transient, material‐dependent immunosuppressive phenotype.^[^ ^168^ ^]^ In an agricultural infection model, AgNPs administered to broiler chickens challenged with *Campylobacter jejuni* reduced weight gain and attenuated immune function, indicating compromised host defense.^[^ ^169^ ^]^ Consistent with these observations, in immunocompetent mice, 28‐day oral exposure to silver colloid decreased peripheral monocyte counts, reduced NK/NKT cell proportions, and suppressed lymphocyte proliferation, suggesting systemic immunosuppression even in the absence of deliberate infection.^[^ ^170^ ^]^ Long‐term safety also varies by material class. Biodegradable carriers (e.g., PLGA, liposomes) clear via metabolism (PLGA to lactic and glycolic acid) and have a favorable clinical track record, although repeated dosing still warrants longitudinal monitoring for microenvironment changes.^[^ ^171^ ^]^ In contrast, nonbiodegradable inorganic NPs (e.g., gold) can persist in reticuloendothelial organs for months, with divergent outcomes across studies: some reports describe long‐term accumulation with minimal overt toxicity (up to 7–15 months in mice), whereas others link chronic retention (e.g., BSA‐coated AuNPs retained for more than 120 days) to early inflammatory and fibrotic responses that are coating‐ and context‐dependent.^[^ ^172^, ^173^, ^174^ ^]^ These data support a material‐informed risk management strategy. Biodegradable platforms should be prioritized for chronic or repeat dosing; if biopersistent materials are required, preclinical evaluation should incorporate exposure ceilings, RES‐organ burden tracking, and fibrosis/inflammation biomarkers, with incidence metrics (e.g., survival, organ fibrosis scoring) reported alongside mechanistic immunoprofiling to enable cross‐study comparison.

Similarly, contaminants such as bacterial endotoxin (LPS) can absorb to NPs during manufacturing, because the high surface area of many NPs allows them to act as “sponges” for endotoxin. Undetected endotoxin can cause potent immune activation and confound results. In fact, more than one third of investigational nanomedicines failed early preclinical testing due to endotoxin contamination issues.^[^ ^175^, ^176^ ^]^ Rigorous endotoxin testing and removal are thus mandatory from a safety standpoint. However, standard Limulus amebocyte lysate (LAL) tests for endotoxin can be confounded by NP interference, sometimes yielding false positives or negatives.^[^ ^177^, ^178^ ^]^ Developers must use multiple orthogonal assays to ensure their product is truly endotoxin‐free.

In summary, developers of Treg‐targeting nanotherapies must navigate a fine line: the NP should be immunologically inert enough not to cause undue activation or allergic reactions, yet effective enough to modulate the immune response in the desired way. Preclinical studies should include extensive immune profiling of Tregs and nontarget leukocyte compartments (e.g., effector T cells and B cells), together with systemic safety readouts such as cytokine surges, complement activation, anticarrier antibodies, and quantitative indices of systemic immunosuppression, to identify immunotoxic liabilities early and reduce the risk of subsequent clinical failure.

### 5.3 Manufacturing and Standardization

Translating nanomedicine from the lab to the clinic requires overcoming significant manufacturing and quality control challenges. NPs are multicomponent systems (comprising core materials, surface coatings, therapeutic payloads, etc.). Small variations in their physicochemical properties can lead to large differences in biological behavior.^[^ ^179^ ^]^ Ensuring batch‐to‐batch consistency is therefore crucial. A slight change in average particle size during scale‐up might alter biodistribution–for instance, a tendency for larger particles to be sequestered by the liver more than intended. Major hurdles for launching a polymeric nanocarrier product include the pronounced sensitivity of PLGA systems to mixing and solvent‐exchange conditions, which frequently manifests as batch‐to‐batch variability during scale‐up.^[^ ^180^ ^]^ Thus, scaled‐up production of NPs must tightly control parameters such as particle size distribution, surface ligand density, and drug loading content.^[^ ^181^, ^182^, ^183^ ^]^ A process‐intensified inline‐sonication, continuous‐processing route combined with continuous‐flow filtration/tangential‐flow filtration, has yielded uniform ≈150 nm PLGA NPs at industrial scale and markedly improved lot‐to‐lot consistency.^[^ ^184^ ^]^ In parallel, computational tools such as machine learning are being explored to streamline NP development and predict critical quality attributes (CQAs), which may further aid in achieving scalable, reproducible manufacturing.^[^ ^185^ ^]^

Another practical bottleneck is storage and distribution. mRNA LNPs generally require an ultra‐cold supply chain (often −20 °C to −80 °C) because both mRNA and lipid components degrade at ambient temperatures, complicating global deployment.^[^ ^186^ ^]^ Lyophilization (freeze‐drying) is an increasingly validated workaround: nucleoside‐modified mRNA‐LNPs retained activity for more than 12 weeks at room temperature and more than 24 weeks at 4 °C after lyophilization, substantially reducing cold‐chain burden.^[^ ^187^ ^]^

Standardization of functional assays is a prerequisite for comparing Treg‐modulating nanomedicines across different platforms, similar to release testing for CAR‐Tregs couples analytical CQA metrics (e.g., CAR expression, viability) with potency assays that measure antigen‐specific suppression.^[^ ^188^, ^189^ ^]^ By analogy, NP products should be released against a harmonized panel that quantifies (i) target engagement (e.g., binding to human Treg‐selective complexes such as GARP‐LAP and their competitive displacement by soluble ligands), (ii) functional readouts of Treg biology (FOXP3 stability, STAT5/IL‐2 signaling competence, epigenetic TSDR demethylation),^[^ ^190^ ^]^ and (iii) systems‐level potency (suppression of effector T cell proliferation/cytokines in standardized coculture or organoid models). As observed with CAR‐Treg constructs, in which the scFv affinity, spacer/hinge, and intracellular signaling domains are tightly controlled to ensure in vivo persistence and safety, Treg‐targeted NPs that incorporate labile biologics (antibodies, scFv fragments, IL‐2 muteins, and cytokines) will likewise require Good Manufacturing Practice (GMP) controls.

### 5.4 Future Opportunities—Single‐Cell Sequencing, CAR‐Tregs, Gene Editing, and Beyond

The future of Treg‐targeted therapy offers possibilities that extend beyond conventional drug delivery. Nanotechnology is poised to converge with high‐throughput sequencing, cell therapy, and gene engineering to create novel therapeutic modalities.

Single‐cell and spatial omics promise to personalize Treg‐targeted nanomedicine by resolving, at patient level, the phenotypic and functional heterogeneity of Tregs and their niches.^[^ ^191^ ^]^ Single‐cell RNA sequencing (scRNA‐seq), CITE‐seq, and scATAC‐seq delineate tissue‐ and disease‐specific Treg states, uncovering actionable surface receptors (e.g., CCR8, CTLA‐4, and TIGIT) and metabolic or cytokine circuits (IL‐2/IL‐2Rα, TGF‐β, and IL‐10) that can guide the selection of NP ligands, cargos, and release strategies with higher selectivity and reduced off‐target immunosuppression. Spatial transcriptomics further maps Treg‐APC‐stromal neighborhoods in inflamed tissues and tumors, informing the route of administration and materials choices (size, charge, stiffness) needed to access privileged microanatomical compartments. These omic platforms also serve as pharmacodynamic companions, monitoring Treg stability (FOXP3, IL2RA, and CTLA4), lineage imprinting, and suppressive programs after administration of cytokine‐, AhR‐agonist‐, or rapamycin‐loaded carriers, while exposing compensatory pathways that can be countered with rational combination therapy. In oncology, scRNA‐seq‐resolved intratumoral Treg states inform dual‐payload designs that preserve effector T cells while compromising Treg fitness (e.g., interference with lactate or TGF‐β pathway interference) and facilitate adaptive scheduling with vaccines or checkpoint blockade.^[^ ^192^, ^193^ ^]^

CAR‐Tregs represent an emerging cell therapy in which patient‐derived Tregs are engineered to express a CAR specific for a given antigen (e.g., a common HLA mismatch in transplantation).^[^ ^194^ ^]^ Early preclinical studies have shown that CAR‐Tregs can suppress immune responses in an antigen‐specific manner more efficiently than polyclonal Tregs.^[^ ^195^, ^196^ ^]^ A first‐in‐human trial of CAR‐Tregs is already underway in kidney transplant patients (Sangamo's TX200 product, which uses a CAR against HLA‐A2).^[^ ^197^ ^]^ Nanotechnology can support CAR‐Treg development in several ways. The manufacture of CAR‐Tregs involves gene delivery to T cells (traditionally via viral vectors); NPs could provide a nonviral alternative for transfecting Tregs with CAR constructs or supporting genes. Recent work have shown that lipid NPs can deliver mRNA encoding CARs to T cells either ex vivo^[^ ^198^ ^]^ or in vivo,^[^ ^199^ ^]^ producing CAR‐T cells without viral vectors and reducing the risk of exhaustion through transient expression.^[^ ^200^ ^]^ Similar approaches could be applied to Tregs, either ex vivo or directly in vivo, to convert Tconvs into CAR‐Tregs at the site of need.

Moreover, NPs can be used as “backpack” delivery systems for cell therapies. For instance, researchers have attached cytokine‐loaded NPs to the surface of T cells to provide sustained signals. In one study, engineered Tregs with TCR‐activated, redox‐sensitive IL‐2 NP conjugates that release IL‐2 in response to T cell activation, thereby enhancing the Treg survival and function after adoptive transfer.^[^ ^85^ ^]^ These kinds of smart NP attachments could increase CAR‐Treg therapies potent by ensuring the infused Tregs stay alive and suppressive in the inflammatory environment of a transplant or autoimmune flare.

Gene therapy offers the tantalizing possibility of permanently enhancing Treg number or function. Applications include diseases of Treg deficiency, such as IPEX syndrome.^[^ ^201^ ^]^ CRISPR‐Cas9 gene editing has recently been used to correct *FOXP3* mutations in patient‐derived cells. Goodwin et al. showed that inserting a correct *FOXP3* cDNA into the endogenous locus of IPEX patient T cells and stem cells restored normal *FOXP3* expression and partial suppressive activity.^[^ ^202^ ^]^ In the future, one could envision using targeted lipid NPs or viral vectors to deliver such gene‐editing components to patient T cells, in order to repair Tregs in vivo or generate new Tregs from naïve T cells.

Another frontier is epigenetic reprogramming of T cells into Tregs. Investigators have attempted to convert Tconvs to iTregs by delivering transcription factors or activating *FOXP3* through CRISPR in naïve T cells. If NPs could deliver mRNAs or transcription factor proteins that push T cells towards a Treg fate, it might be possible to induce tolerance in situ without needing to transfer cells. For instance, Honaker et al. recently described an approach to enforce *FOXP3* expression in primary human CD4^+^ T cells via CRISPR‐mediated insertion of a strong promoter, essentially creating suppressive T cells from ordinary T cells.^[^ ^203^ ^]^ While these approaches are at an early stage, nanocarriers for selective delivery of such gene‐modifying tools to the relevant T cell subsets would be game‐changing.

### 5.5 Nobel‐Inspired Perspective

The 2025 Nobel Prize in Physiology or Medicine, awarded to Brunkow, Ramsdell, and Sakaguchi for discovering peripheral immune tolerance, underscores the foundational role of Tregs in immune homeostasis. Peripheral immune tolerance has moved from a descriptive concept to a clinically actionable design principle. The field is now positioned to build medicines that do not treat the immune system as globally overactive or globally exhausted but instead recalibrate defined circuits of control. The ability of NPs to codeliver ligands, cytokine payloads, or gene‐regulatory constructs to immunologically privileged niches makes this selective pressure technically achievable and conceptually aligned with the tolerance framework recognized by the Prize. We anticipate that merging Treg biology with nanomaterial engineering will enable therapies that can induce antigen‐focused immune calm in autoimmunity and transplantation and conversely relieve pathologic immune suppression in cancer, without defaulting to blunt systemic immunosuppression or permanent immune depletion. In our view, this convergence is likely to set the template for a next generation of tolerance‐inducing and tolerance‐relieving interventions that are precise, modular, and clinically scalable.

## Conflict of Interest

The authors declare no conflict of interest.

## References

[1] S. Sakaguchi , T. Yamaguchi , T. Nomura , M. Ono , Cell 2008, 133, 775. 18510923 10.1016/j.cell.2008.05.009

[2] X. Huang , J. Zhu , Y. Yang , J. Immunol. 2005, 175, 4283. 16177068 10.4049/jimmunol.175.7.4283

[3] C. Tay , A. Tanaka , S. Sakaguchi , Cancer Cell. 2023, 41, 450. 36917950 10.1016/j.ccell.2023.02.014

[4] K. Jh , Trends Cancer 2023, 9.

[5] B. S. Zolnik , A. González‐Fernández , N. Sadrieh , M. A. Dobrovolskaia , Endocrinology 2010, 151, 458. 20016026 10.1210/en.2009-1082 PMC2817614

[6] D. M. Smith , J. K. Simon , J. R. Baker , Nat. Rev. Immunol. 2013, 13, 592. 23883969 10.1038/nri3488 PMC7097370

[7] K. R. Rhodes , R. A. Meyer , J. Wang , S. Y. Tzeng , J. J. Green , Acta Biomater. 2020, 112, 136. 32522714 10.1016/j.actbio.2020.06.004 PMC7365762

[8] R. A. Maldonado , R. A. LaMothe , J. D. Ferrari , A. H. Zhang , R. J. Rossi , P. N. Kolte , A. P. Griset , C. O’Neil , D. H. Altreuter , E. Browning , L. Johnston , O. C. Farokhzad , R. Langer , D. W. Scott , U. H. von Andrian , T. K. Kishimoto , Proc. Natl. Acad. Sci. 2015, 112, E156. 25548186 10.1073/pnas.1408686111 PMC4299193

[9] M. Haist , V. Mailänder , M. Bros , Front. Immunol. 2022, 13, 912594. 35693776 10.3389/fimmu.2022.912594 PMC9174908

[10] D. R. Getts , L. D. Shea , S. D. Miller , N. J. C. King , Trends Immunol. 2015, 36, 419. 26088391 10.1016/j.it.2015.05.007 PMC4603374

[11] A. Zhang , T. Fan , Y. Liu , G. Yu , C. Li , Z. Jiang , Mol. Cancer 2024, 23, 251. 39516941 10.1186/s12943-024-02156-y PMC11545879

[12] H. J. J. van der Vliet , E. E. Nieuwenhuis , Clin. Dev. Immunol. 2007, 2007, 89017. 18317533 10.1155/2007/89017 PMC2248278

[13] P. A. Savage , D. E. J. Klawon , C. H. Miller , Annu. Rev. Immunol. 2020, 38, 421. 31990619 10.1146/annurev-immunol-100219-020937

[14] D. L. Owen , L. E. Sjaastad , M. A. Farrar , J. Immunol. 2019, 203, 2031. 31591259 10.4049/jimmunol.1900662 PMC6910132

[15] S. Hori , T. Nomura , S. Sakaguchi , Science 2003, 299, 1057. 28115586

[16] J. D. Fontenot , M. A. Gavin , A. Y. Rudensky , Nat. Immunol. 2003, 4, 330. 28115587

[17] M. S. Jordan , A. Boesteanu , A. J. Reed , A. L. Petrone , A. E. Holenbeck , M. A. Lerman , A. Naji , A. J. Caton , Nat. Immunol. 2001, 2, 301. 11276200 10.1038/86302

[18] J. L. Coombes , K. R. R. Siddiqui , C. V. Arancibia‐Cárcamo , J. Hall , C.‐M. Sun , Y. Belkaid , F. Powrie , J. Exp. Med. 2007, 204, 1757. 17620361 10.1084/jem.20070590 PMC2118683

[19] G. Bakdash , L. T. C. Vogelpoel , T. M. M. van Capel , M. L. Kapsenberg , E. C. de Jong , Mucosal Immunol. 2015, 8, 265. 25027601 10.1038/mi.2014.64

[20] T. Chinen , A. K. Kannan , A. G. Levine , X. Fan , U. Klein , Y. Zheng , G. Gasteiger , Y. Feng , J. D. Fontenot , A. Y. Rudensky , Nat. Immunol. 2016, 17, 1322. 27595233 10.1038/ni.3540 PMC5071159

[21] R. Opstelten , S. de Kivit , M. C. Slot , M. van den Biggelaar , D. Iwaszkiewicz‐Grześ , M. Gliwiński , A. M. Scott , B. Blom , P. Trzonkowski , J. Borst , E. Cuadrado , D. Amsen , J. Immunol. 2020, 204, 3139. 32366581 10.4049/jimmunol.1901250

[22] D. A. A. Vignali , L. W. Collison , C. J. Workman , Nat. Rev. Immunol. 2008, 8, 523. 18566595 10.1038/nri2343 PMC2665249

[23] L. W. Collison , C. J. Workman , T. T. Kuo , K. Boyd , Y. Wang , K. M. Vignali , R. Cross , D. Sehy , R. S. Blumberg , D. A. A. Vignali , Nature 2007, 450, 566. 18033300 10.1038/nature06306

[24] P. Pandiyan , L. Zheng , S. Ishihara , J. Reed , M. J. Lenardo , Nat. Immunol. 2007, 8, 1353. 17982458 10.1038/ni1536

[25] F. Fallarino , U. Grohmann , K. W. Hwang , C. Orabona , C. Vacca , R. Bianchi , M. L. Belladonna , M. C. Fioretti , M.‐L. Alegre , P. Puccetti , Nat. Immunol. 2003, 4, 1206. 14578884 10.1038/ni1003

[26] C. T. Huang , C. J. Workman , D. Flies , X. Pan , A. L. Marson , G. Zhou , E. L. Hipkiss , S. Ravi , J. Kowalski , H. I. Levitsky , J. D. Powell , D. M. Pardoll , C. G. Drake , D. A. A. Vignali , Immunity 2004, 21, 503. 15485628 10.1016/j.immuni.2004.08.010

[27] N. Joller , E. Lozano , P. R. Burkett , B. Patel , S. Xiao , C. Zhu , J. Xia , T. G. Tan , E. Sefik , V. Yajnik , A. H. Sharpe , F. J. Quintana , D. Mathis , C. Benoist , D. A. Hafler , V. K. Kuchroo , Immunity 2014, 40, 569. 24745333 10.1016/j.immuni.2014.02.012 PMC4070748

[28] W. J. Grossman , J. W. Verbsky , W. Barchet , M. Colonna , J. P. Atkinson , T. J. Ley , Immunity 2004, 21, 589. 15485635 10.1016/j.immuni.2004.09.002

[29] D. C. Gondek , L. F. Lu , S. A. Quezada , S. Sakaguchi , R. J. Noelle , J. Immunol. 2005, 174, 1783. 15699103 10.4049/jimmunol.174.4.1783

[30] X. Cao , S. F. Cai , T. A. Fehniger , J. Song , L. I. Collins , D. R. Piwnica‐Worms , T. J. Ley , Immunity 2007, 27, 635. 17919943 10.1016/j.immuni.2007.08.014

[31] S. Deaglio , K. M. Dwyer , W. Gao , D. Friedman , A. Usheva , A. Erat , J.‐F. Chen , K. Enjyoji , J. Linden , M. Oukka , V. K. Kuchroo , T. B. Strom , S. C. Robson , J. Exp. Med. 2007, 204, 1257. 17502665 10.1084/jem.20062512 PMC2118603

[32] M. A. Farooq , A. P. R. Johnston , N. L. Trevaskis , Acta Biomater. 2025, 193, 65. 39701340 10.1016/j.actbio.2024.12.039

[33] D. A. Boardman , M. K. Levings , J. Allergy Clin. Immunol. 2022, 149, 1. 34998473 10.1016/j.jaci.2021.11.007

[34] N. Benne , D. Ter Braake , A. J. Stoppelenburg , F. Broere , Front. Immunol. 2022, 13, 864403. 35392079 10.3389/fimmu.2022.864403 PMC8981588

[35] S. Singha , K. Shao , Y. Yang , X. Clemente‐Casares , P. Solé , A. Clemente , J. Blanco , Q. Dai , F. Song , S. W. Liu , J. Yamanouchi , C. S. Umeshappa , R. H. Nanjundappa , P. Detampel , M. Amrein , C. Fandos , R. Tanguay , S. Newbigging , P. Serra , A. Khadra , W. C. W. Chan , P. Santamaria , Nat. Nanotechnol. 2017, 12, 701. 28436959 10.1038/nnano.2017.56

[36] D. R. Getts , A. J. Martin , D. P. McCarthy , R. L. Terry , Z. N. Hunter , W. T. Yap , M. T. Getts , M. Pleiss , X. Luo , N. J. C. King , L. D. Shea , S. D. Miller , Nat. Biotechnol. 2012, 30, 1217. 23159881 10.1038/nbt.2434 PMC3589822

[37] Q. Chen , Y. C. Kim , A. Laurence , G. A. Punkosdy , E. M. Shevach , J. Immunol. 2011, 186, 6329. 21525380 10.4049/jimmunol.1100061 PMC3098943

[38] S. G. Zheng , J. Wang , P. Wang , J. D. Gray , D. A. Horwitz , J. Immunol. 2007, 178, 2018. 17277105 10.4049/jimmunol.178.4.2018

[39] J. H. Donohue , S. A. Rosenberg , J. Immunol. 1983, 130, 2203. 6601147

[40] M. D. McHugh , J. Park , R. Uhrich , W. Gao , D. A. Horwitz , T. M. Fahmy , Biomaterials 2015, 59, 172. 25974747 10.1016/j.biomaterials.2015.04.003 PMC5997248

[41] D. A. Horwitz , S. Bickerton , M. Koss , T. M. Fahmy , A. La Cava , Arthritis Rheumatol. 2019, 71, 632. 30407752 10.1002/art.40773 PMC6438734

[42] H. Li , M. G. Tsokos , S. Bickerton , A. Sharabi , Y. Li , V. R. Moulton , P. Kong , T. M. Fahmy , G. C. Tsokos , JCI Insight. 2018, 3, e120880. 30135300 10.1172/jci.insight.120880 PMC6141184

[43] S. Floess , J. Freyer , C. Siewert , U. Baron , S. Olek , J. Polansky , K. Schlawe , H.‐D. Chang , T. Bopp , E. Schmitt , S. Klein‐Hessling , E. Serfling , A. Hamann , J. Huehn , PLoS Biol. 2007, 5, e38. 17298177 10.1371/journal.pbio.0050038 PMC1783672

[44] J. K. Polansky , K. Kretschmer , J. Freyer , S. Floess , A. Garbe , U. Baron , S. Olek , A. Hamann , H. von Boehmer , J. Huehn , Eur. J. Immunol. 2008, 38, 1654. 18493985 10.1002/eji.200838105

[45] D. Gallo , D. Baci , N. Kustrimovic , N. Lanzo , B. Patera , M. L. Tanda , E. Piantanida , L. Mortara , Int. J. Mol. Sci. 2023, 24, 4689. 36902117 10.3390/ijms24054689 PMC10003699

[46] G. Lin , J. Wang , Y. G. Yang , Y. Zhang , T. Sun , Front. Bioeng. Biotechnol. 2023, 11, 1242126. 37877041 10.3389/fbioe.2023.1242126 PMC10593475

[47] H. H. Jung , S. H. Kim , J. H. Moon , S. U. Jeong , S. Jang , C. S. Park , C. K. Lee , Immune Netw. 2019, 19, e19. 31281716 10.4110/in.2019.19.e19 PMC6597444

[48] J. M. Gammon , L. H. Tostanoski , A. R. Adapa , Y. C. Chiu , C. M. Jewell , J. Control Release 2015, 210, 169. 26002150 10.1016/j.jconrel.2015.05.277

[49] J. M. Gammon , A. R. Adapa , C. M. Jewell , J. Biomed. Mater. Res. A. 2017, 105, 2977. 28646511 10.1002/jbm.a.36151

[50] C. Engman , Y. Wen , W. S. Meng , R. Bottino , M. Trucco , N. Giannoukakis , Clin. Immunol. 2015, 160, 103. 25773782 10.1016/j.clim.2015.03.004

[51] X. Tang , Y. Shang , H. Yang , Song , S. Li , Y. Qin , J. Song , K. Chen , Y. Liu , D. Zhang , L. Chen , Nat. Commun. 2024, 15, 1673. 38396052 10.1038/s41467-024-46025-0 PMC10891058

[52] T. Yokosuka , M. Takamatsu , W. Kobayashi‐Imanishi , A. Hashimoto‐Tane , M. Azuma , T. Saito , J. Exp. Med. 2012, 209, 1201. 22641383 10.1084/jem.20112741 PMC3371732

[53] S. Amarnath , C. W. Mangus , J. C. M. Wang , F. Wei , A. He , V. Kapoor , J. E. Foley , P. R. Massey , T. C. Felizardo , J. L. Riley , B. L. Levine , C. H. June , J. A. Medin , D. H. Fowler , Sci. Transl. Med. 2011, 3, 111ra120. 10.1126/scitranslmed.3003130 PMC3235958 22133721

[54] L. M. Francisco , V. H. Salinas , K. E. Brown , V. K. Vanguri , G. J. Freeman , V. K. Kuchroo , A. H. Sharpe , J. Exp. Med. 2009, 206, 3015. 20008522 10.1084/jem.20090847 PMC2806460

[55] Y. L. Luo , L. F. Liang , Y. J. Gan , J. Liu , Y. Zhang , Y. N. Fan , G. Zhao , A. Czarna , Z. D. Lu , X. J. Du , S. Shen , C. F. Xu , Z. X. Lian , J. Wang , ACS Appl. Mater. Interfaces 2020, 12, 48259. 33070614 10.1021/acsami.0c10885

[56] Y. Liu , Q. Liu , B. Zhang , Z. Li , S. Zhou , Y. Liu , X. Zhou , C. Wang , C. Wang , J. Wang , Nat. Biomed. Eng. 2025, 9, 1320. 40155762 10.1038/s41551-025-01373-0

[57] J. Li , W. Hou , S. Lin , Y. Yu , H. Ji , N. Li , Y. Li , Z. Y. Ong , P. Gan , L. Zhu , Y. Zhang , O. Keissler , X. Liu , G. Pastorin , X. Zhang , Adv. Sci. Weinh. 2022, 9, e2104006. 34713621 10.1002/advs.202104006 PMC8728836

[58] C. Capini , M. Jaturanpinyo , H. I. Chang , S. Mutalik , A. McNally , S. Street , R. Steptoe , B. O’Sullivan , N. Davies , R. Thomas , J. Immunol. 2009, 182, 3556. 19265134 10.4049/jimmunol.0802972

[59] S. Sauer , L. Bruno , A. Hertweck , D. Finlay , M. Leleu , M. Spivakov , Z. A. Knight , B. S. Cobb , D. Cantrell , E. O’Connor , K. M. Shokat , A. G. Fisher , M. Merkenschlager , Proc. Natl. Acad. Sci. 2008, 105, 7797. 18509048 10.1073/pnas.0800928105 PMC2409380

[60] R. A. LaMothe , P. N. Kolte , T. Vo , J. D. Ferrari , T. C. Gelsinger , J. Wong , V. T. Chan , S. Ahmed , A. Srinivasan , P. Deitemeyer , R. A. Maldonado , T. K. Kishimoto , Front. Immunol. 2018, 9, 281. 29552007 10.3389/fimmu.2018.00281 PMC5840162

[61] X. Chen , G. Du , S. Bai , D. Li , C. Li , Y. Hou , Y. Zhang , Y. Zhang , T. Gong , Y. Fu , M. Bottini , X. Sun , Nano Today. 2021, 41, 101307.

[62] X. Zhang , D. Liu , M. He , M. Lin , C. Tu , B. Zhang , Hum. Vac. Immunother. 2021, 17, 1923. 10.1080/21645515.2021.1872342 PMC8189063 33616474

[63] T. K. Kishimoto , M. Fournier , A. Michaud , G. Rizzo , C. Roy , T. Capela , N. Nukolova , N. Li , L. Doyle , F. N. Fu , D. VanDyke , P. G. Traber , J. B. Spangler , S. S. Leung , P. O. Ilyinskii , J. Autoimmun. 2023, 140, 103125. 37844543 10.1016/j.jaut.2023.103125

[64] J. A. Goettel , R. Gandhi , J. E. Kenison , A. Yeste , G. Murugaiyan , S. Sambanthamoorthy , A. E. Griffith , B. Patel , D. S. Shouval , H. L. Weiner , S. B. Snapper , F. J. Quintana , Cell Rep. 2016, 17, 1318. 27783946 10.1016/j.celrep.2016.09.082 PMC5106873

[65] Q. Zhang , Y. Zhu , C. Lv , Y. Fang , M. Liao , Y. Xia , Z. Wei , Y. Dai , Immunology 2023, 169, 412. 36930164 10.1111/imm.13638

[66] A. F. David , A. Heinzel , M. Kammer , S. Enengl , E. Salzer , M. T. Lindenmeyer , J. Nilsson , H. Himmelreich , T. Nothnagl , C. H. Shen , M. Ortner , G. Jeschke , J. B. Huppa , A. Barta , F. Fresser , M. Niederberger , J. Thome , G. Superti‐Furga , M. C. A. Karlsson , S. Freunberger , C. B. Schönlieb , EBioMedicine 2024, 106, 105239. 38996766 10.1016/j.ebiom.2024.105239 PMC11284950

[67] B. Sawitzki , P. N. Harden , P. Reinke , A. Moreau , J. A. Hutchinson , D. S. Game , Q. Tang , E. C. Guinan , M. Battaglia , W. J. Burlingham , I. S. D. Roberts , M. Streitz , R. Josien , C. A. Böger , C. Scottà , J. F. Markmann , J. L. Hester , K. Juerchott , C. Braudeau , B. James , L. Contreras-Ruiz , J. B. van der Net , T. Bergler , R. Caldara , W. Petchey , M. Edinger , N. Dupas , M. Kapinsky , I. Mutzbauer , N. M. Otto , et al., Lancet 2020, 395, 1627. 32446407 10.1016/S0140-6736(20)30167-7 PMC7613154

[68] A. Yeste , M. C. Takenaka , I. D. Mascanfroni , M. Nadeau , J. E. Kenison , B. Patel , A. M. Tukpah , J. A. B. Babon , M. DeNicola , S. C. Kent , D. Pozo , F. J. Quintana , Sci Signal. 2016, 9, ra61. 27330188 10.1126/scisignal.aad0612

[69] J. E. Kenison , A. Jhaveri , Z. Li , S. I. Grivennikov , M. Karin , D. A. A. Vignali , D. A. Carson , M. Downes , R. M. Evans , D. H. Conrad , H. L. Weiner , F. J. Quintana , Proc. Natl. Acad. Sci. 2020, 117, 32017.33239445

[70] G. Coutance , E. Desiré , J. P. Duong Van Huyen , Biomolecules 2022, 12, 1135. 36009029 10.3390/biom12081135 PMC9405997

[71] R. Zeiser , B. R. Blazar , N. Engl. J. Med. 2017, 377, 2167. 29171820 10.1056/NEJMra1609337 PMC6034180

[72] M. Di Ianni , F. Falzetti , A. Carotti , A. Terenzi , F. Castellino , E. Bonifacio , B. Del Papa , T. Zei , R. Iacucci Ostini , D. Cecchini , T. Aloisi , K. Perruccio , L. Ruggeri , C. Balucani , A. Pierini , P. Sportoletti , C. Aristei , B. Falini , Y. Reisner , A. Velardi , F. Aversa , M. F. Martelli , Blood 2011, 117, 3921. 21292771 10.1182/blood-2010-10-311894

[73] C. G. Brunstein , J. S. Miller , D. H. McKenna , K. L. Hippen , T. E. DeFor , D. Sumstad , J. Curtsinger , M. R. Verneris , M. L. MacMillan , B. L. Levine , J. L. Riley , C. H. June , C. Le , D. J. Weisdorf , P. B. McGlave , B. R. Blazar , J. E. Wagner , Blood 2016, 127, 1044. 26563133 10.1182/blood-2015-06-653667 PMC4768428

[74] C. S. Bader , A. Pavlova , R. Lowsky , L. S. Muffly , P. Shiraz , S. Arai , L. J. Johnston , A. R. Rezvani , W.‐K. Weng , D. B. Miklos , M. J. Frank , J. S. Tamaresis , V. Agrawal , S. Bharadwaj , S. Sidana , J. A. Shizuru , N. B. Fernhoff , A. Putnam , S. Killian , B. J. Xie , R. S. Negrin , E. H. Meyer , Blood Adv. 2024, 8, 1105. 38091578 10.1182/bloodadvances.2023011625 PMC10907400

[75] E. H. Meyer , A. Pavlova , A. Villar‐Prados , C. Bader , B. Xie , L. Muffly , P. Kim , K. Sutherland , S. Bharadwaj , S. Dahiya , M. Frank , S. Arai , L. Johnston , D. Miklos , A. Rezvani , P. Shiraz , S. Sidana , J. Shizuru , W.‐K. Weng , V. Agrawal , A. Putnam , N. Fernhoff , J. Tamarisis , Y. Lu , R. D. Pawar , J. S. McClellan , R. Lowsky , R. S. Negrin , Blood 2025, 145, 2012. 39792934 10.1182/blood.2024026446 PMC12782974

[76] S. Shah , S. Daneshmandi , K. R. Hughes , S. Yu , A. M. Bedoya , L. D. Shea , X. Luo , Biomaterials 2019, 210, 70. 31077862 10.1016/j.biomaterials.2019.04.030 PMC6528823

[77] J. Song , J. Huang , X. Chen , X. Teng , Z. Song , Y. Xing , M. Wang , K. Chen , Z. Wang , P. Yang , S. Hu , Sci. Rep. 2016, 7, 20077. 26822278 10.1038/srep20077 PMC4731812

[78] J. Bryant , K. A. Hlavaty , X. Zhang , W. T. Yap , L. Zhang , L. D. Shea , X. Luo , Biomaterials 2014, 35, 8887. 25066477 10.1016/j.biomaterials.2014.06.044 PMC4231141

[79] K. Xing , Y. Che , Z. Wang , S. Yuan , Q. Wu , F. Shi , Y. Chen , X. Shen , X. Zhong , X. Xie , Q. Zhu , X. Li , Int. Immunopharmacol. 2023, 124, 110922. 37699303 10.1016/j.intimp.2023.110922

[80] P. S. Randhawa , T. E. Starzl , A. J. Demetris , Adv. Anat. Pathol. 1997, 4, 265. 21572890 10.1097/00125480-199707000-00032 PMC3092646

[81] Q. Zeng , X. Y. Yuan , W. Li , B. W. Liu , X. Zhao , G. J. Ren , Y. Wang , J. Dou , G. Y. Wang , Immunopharmacol. Immunotoxicol. 2019, 41, 380. 30633591 10.1080/08923973.2018.1533026

[82] C. Miroux , O. Morales , K. Ghazal , S. Ben Othman , Y. de Launoit , V. Pancré , F. Conti , N. Delhem , Transplantation 2012, 94, 123. 22743548 10.1097/TP.0b013e3182590d8f

[83] Z. Cao , C. Li , J. He , X. Sui , P. Wu , D. Pan , L. Qing , J. Tang , Ann. Transl. Med. 2021, 9, 1515. 34790721 10.21037/atm-21-2425 PMC8576731

[84] D. A. Horwitz , J. H. Wang , D. Kim , C. Kang , K. Brion , S. Bickerton , A. La Cava , Front. Immunol. 2024, 15, 1429335. 39131162 10.3389/fimmu.2024.1429335 PMC11310063

[85] G. B. Hamra , N. Guha , A. Cohen , F. Laden , O. Raaschou‐Nielsen , J. M. Samet , P. Vineis , F. Forastiere , P. Saldiva , T. Yorifuji , D. Loomis , Environ. Health Perspect. 2014, 122, 906. 24911630 10.1289/ehp/1408092 PMC4154221

[86] Y. Zhang , S. Shen , G. Zhao , C. F. Xu , H. B. Zhang , Y. L. Luo , Z. T. Cao , J. Shi , Z. B. Zhao , Z. X. Lian , J. Wang , Biomaterials 2019, 217, 119302. 31271858 10.1016/j.biomaterials.2019.119302

[87] B. Bahmani , M. Uehara , L. Jiang , F. Ordikhani , N. Banouni , T. Ichimura , Z. Solhjou , G. J. Furtmüller , G. Brandacher , D. Alvarez , U. H. von Andrian , K. Uchimura , Q. Xu , I. Vohra , O. A. Yilmam , Y. Haik , J. Azzi , V. Kasinath , J. S. Bromberg , M. M. McGrath , R. Abdi , J. Clin. Invest. 2018, 128, 4770. 30277476 10.1172/JCI120923 PMC6205374

[88] J. Zhao , S. Jung , X. Li , L. Li , V. Kasinath , H. Zhang , S. N. Movahedi , A. Mardini , G. Sabiu , Y. Hwang , V. Saxena , Y. Song , B. Ma , S. E. Acton , P. Kim , J. C. Madsen , P. T. Sage , S. G. Tullius , G. C. Tsokos , J. S. Bromberg , R. Abdi , J. Clin. Invest. 2022, 132, e159672. 36519543 10.1172/JCI159672 PMC9754003

[89] R. Zeiser , B. R. Blazar , N. Engl. J. Med. 2017, 377, 2565. 29281578 10.1056/NEJMra1703472

[90] S. Giang , D. A. Horwitz , S. Bickerton , A. La Cava , Front. Immunol. 2021, 12, 628059. 34122401 10.3389/fimmu.2021.628059 PMC8189151

[91] Y. Lee , H. Kim , S. Kang , J. Lee , J. Park , S. Jon , Angew. Chem. Int. Ed. Engl. 2016, 55, 7460. 27144463 10.1002/anie.201602525

[92] S. Pareek , A. S. Flegle , D. Boagni , J. Y. Kim , D. Yoo , A. Trujillo‐Ocampo , S.‐E. Lee , M. Zhang , S. Jon , J. S. Im , Front. Immunol. 2022, 13, 893659. 35720391 10.3389/fimmu.2022.893659 PMC9199387

[93] C. Liu , M. Chikina , R. Deshpande , A. V. Menk , T. Wang , T. Tabib , E. A. Brunazzi , K. M. Vignali , M. Sun , D. B. Stolz , R. A. Lafyatis , W. Chen , G. M. Delgoffe , C. J. Workman , S. G. Wendell , D. A. A. Vignali , Immunity 2019, 51, 381. 31350177 10.1016/j.immuni.2019.06.017 PMC6703933

[94] Y. Yan , L. Huang , Y. Liu , M. Yi , Q. Chu , D. Jiao , K. Wu , J. Hematol. Oncol. 2022, 15, 104. 35948909 10.1186/s13045-022-01322-3 PMC9364625

[95] A. P. Kohm , J. S. McMahon , J. R. Podojil , W. S. Begolka , M. DeGutes , D. J. Kasprowicz , S. F. Ziegler , S. D. Miller , J. Immunol. 2006, 176, 3301. 16517695 10.4049/jimmunol.176.6.3301

[96] W. Ou , R. K. Thapa , L. Jiang , Z. C. Soe , M. Gautam , J. H. Chang , J. H. Jeong , S. K. Ku , H. G. Choi , C. S. Yong , J. O. Kim , J. Control Release 2018, 281, 84. 29777794 10.1016/j.jconrel.2018.05.018

[97] V. P. Balachandran , M. J. Cavnar , S. Zeng , Z. M. Bamboat , L. M. Ocuin , H. Obaid , E. C. Sorenson , R. Popow , C. Ariyan , F. Rossi , P. Besmer , T. Guo , C. R. Antonescu , T. Taguchi , J. Yuan , J. D. Wolchok , J. P. Allison , R. P. DeMatteo , Nat. Med. 2011, 17, 1094. 21873989 10.1038/nm.2438 PMC3278279

[98] H. Chen , X. Luan , H. J. Paholak , J. P. Burnett , N. O. Stevers , K. Sansanaphongpricha , M. He , A. E. Chang , Q. Li , D. Sun , Nanomed. Lond. 2020, 15, 77. 10.2217/nnm-2019-0190 PMC7132783 31868112

[99] H. Chen , J. Burnett , F. Zhang , J. Zhang , H. Paholak , D. Sun , J. Mater. Chem. B 2014, 2, 757. 32261307 10.1039/c3tb21338b

[100] W. Ou , L. Jiang , R. K. Thapa , Z. C. Soe , K. Poudel , J. H. Chang , S. K. Ku , H.‐G. Choi , C. S. Yong , J. O. Kim , Theranostics 2018, 8, 4574. 30279723 10.7150/thno.26758 PMC6160765

[101] S. Y. Li , Y. Liu , C. F. Xu , S. Shen , R. Sun , X. J. Du , J. X. Xia , Y. H. Zhu , J. Wang , J. Control Release 2016, 231, 17. 26829099 10.1016/j.jconrel.2016.01.044

[102] M. Barati , F. Mirzavi , A. R. Nikpoor , M. Sankian , H. N. Ahmadabad , A. Soleimani , M. Mashreghi , J. T. Afshar , M. Mohammadi , M. R. Jaafari , Cancer Gene Ther. 2022, 29, 814. 34341501 10.1038/s41417-021-00367-9

[103] C. Zhao , C. Wang , R. Wang , W. Shan , W. Wang , H. Deng , ACS Nano 2024, 18, 24105. 39171893 10.1021/acsnano.4c04663

[104] V. L. Payen , E. Mina , V. F. Van Hée , P. E. Porporato , P. Sonveaux , Mol. Metab. 2020, 33, 48. 31395464 10.1016/j.molmet.2019.07.006 PMC7056923

[105] O. R. Colegio , N. Q. Chu , A. L. Szabo , T. Chu , A. M. Rhebergen , V. Jairam , N. Cyrus , C. E. Brokowski , S. C. Eisenbarth , G. M. Phillips , G. W. Cline , A. J. Phillips , R. Medzhitov , Nature 2014, 513, 559. 25043024 10.1038/nature13490 PMC4301845

[106] M. J. Watson , P. D. A. Vignali , S. J. Mullett , A. E. Overacre‐Delgoffe , R. M. Peralta , S. Grebinoski , A. V. Menk , N. L. Rittenhouse , K. DePeaux , R. D. Whetstone , D. A. A. Vignali , T. W. Hand , A. C. Poholek , B. M. Morrison , J. D. Rothstein , S. G. Wendell , G. M. Delgoffe , Nature 2021, 591, 645. 33589820 10.1038/s41586-020-03045-2 PMC7990682

[107] K. Li , C. Lin , Y. He , L. Lu , K. Xu , B. Tao , Z. Xia , R. Zeng , Y. Mao , Z. Luo , K. Cai , ACS Nano 2020, 14, 14164. 32975406 10.1021/acsnano.0c07071

[108] X. Zhu , W. Tang , Z. Fan , S. Sun , X. Tan , Biochim. Biophys. Acta Mol. Basis Dis. 2025, 1871, 167641. 39719204 10.1016/j.bbadis.2024.167641

[109] J. S. Suk , Q. Xu , N. Kim , J. Hanes , L. M. Ensign , Adv. Drug Deliv. Rev. 2016, 99, 28. 26456916 10.1016/j.addr.2015.09.012 PMC4798869

[110] A. N. Ilinskaya , M. A. Dobrovolskaia , Br. J. Pharmacol. 2014, 171, 3988. 24724793 10.1111/bph.12722 PMC4243973

[111] A. A. Aljabali , M. A. Obeid , R. M. Bashatwah , Á. Serrano‐Aroca , V. Mishra , Y. Mishra , M. El‐Tanani , A. Hromić‐Jahjefendić , D. N. Kapoor , R. Goyal , G. A. Naikoo , M. M. Tambuwala , Int. J. Mol. Sci. 2023, 24, 2008. 36768330 10.3390/ijms24032008 PMC9917130

[112] R. Mohamud , J. S. LeMasurier , J. C. Boer , J. L. Sieow , J. M. Rolland , R. E. O’Hehir , C. L. Hardy , M. Plebanski , Front. Immunol. 2017, 8, 1812. 29312323 10.3389/fimmu.2017.01812 PMC5744007

[113] K. Yamashita , M. Sakai , N. Takemoto , M. Tsukimoto , K. Uchida , H. Yajima , S. Oshio , K. Takeda , S. Kojima , Toxicology 2009, 261, 19. 19376187 10.1016/j.tox.2009.04.034

[114] L. A. Mitchell , F. T. Lauer , S. W. Burchiel , J. D. McDonald , Nat. Nanotechnol. 2009, 4, 451. 19581899 10.1038/nnano.2009.151 PMC3641180

[115] F. Blank , P. Gerber , B. Rothen‐Rutishauser , U. Sakulkhu , J. Salaklang , K. De Peyer , P. Gehr , L. P. Nicod , H. Hofmann , T. Geiser , A. Petri‐Fink , C. von Garnier , Nanotoxicology 2011, 5, 606. 21231795 10.3109/17435390.2010.541293

[116] M. A. Boks , J. R. Kager‐Groenland , M. S. P. Haasjes , J. J. Zwaginga , S. M. van Ham , A. ten Brinke , Clin. Immunol. 2012, 142, 332. 22225835 10.1016/j.clim.2011.11.011

[117] A. N. Yilma , S. R. Singh , S. Dixit , V. A. Dennis , Int. J. Nanomed. 2013, 8, 2421. 10.2147/IJN.S44090 PMC3709643 23882139

[118] J. Tian , K. K. Y. Wong , C. M. Ho , C. N. Lok , W. Y. Yu , C. M. Che , J. F. Chiu , P. K. H. Tam , ChemMedChem 2007, 2, 129. 17075952 10.1002/cmdc.200600171

[119] V. V. Sumbayev , I. M. Yasinska , C. P. Garcia , D. Gilliland , G. S. Lall , B. F. Gibbs , D. R. Bonsall , L. Varani , F. Rossi , L. Calzolai , Small 2013, 9, 472. 23112137 10.1002/smll.201201528

[120] C. C. Shen , C. C. Wang , M. H. Liao , T. R. Jan , Int. J. Nanomed. 2011, 6, 1229. 10.2147/IJN.S21019 PMC3131189 21753874

[121] M. B. Mia , R. K. Saxena , Immunol. Lett. 2020, 224, 30. 32504776 10.1016/j.imlet.2020.05.006

[122] M. Wang , F. Yu , Y. Zhang , Mol. Cancer 2025, 24, 26. 39827147 10.1186/s12943-024-02214-5 PMC11748575

[123] S. Zuo , J. Song , J. Zhang , Z. He , B. Sun , J. Sun , Theranostics 2021, 11, 7471. 34158861 10.7150/thno.59953 PMC8210608

[124] F. Chen , Y. Wang , J. Gao , M. Saeed , T. Li , W. Wang , H. Yu , Biomaterials 2021, 270, 120709. 33581608 10.1016/j.biomaterials.2021.120709

[125] H. Yano , L. P. Andrews , C. J. Workman , D. A. A. Vignali , Immunology 2019, 157, 232. 31087644 10.1111/imm.13067 PMC6587321

[126] M. O. Mohsen , R. Josi , S. V. Marar , A. Ghimire , L. Yang , P. S. Krenger , A. Solé Casaramona , D. E. Speiser , S. De Brot , M. F. Bachmann , NPJ Vac. 2025, 10, 125. 10.1038/s41541-025-01177-y PMC12166054 40514382

[127] D. Cai , W. Gao , Z. Li , Y. Zhang , L. Xiao , Y. Xiao , Biomedicines 2022, 10, 1203. 35625939 10.3390/biomedicines10051203 PMC9139084

[128] C. Sacchetti , N. Rapini , A. Magrini , E. Cirelli , S. Bellucci , M. Mattei , N. Rosato , N. Bottini , M. Bottini , Bioconjug. Chem. 2013, 24, 852. 23682992 10.1021/bc400070q

[129] N. Seddiki , B. Santner‐Nanan , J. Martinson , J. Zaunders , S. Sasson , A. Landay , M. Solomon , W. Selby , S. I. Alexander , R. Nanan , A. Kelleher , B. Fazekas de St Groth , J. Exp. Med. 2006, 203, 1693. 16818676 10.1084/jem.20060468 PMC2118333

[130] K. Schuh , T. Twardzik , B. Kneitz , J. Heyer , A. Schimpl , E. Serfling , J. Exp. Med. 1998, 188, 1369. 9763616 10.1084/jem.188.7.1369 PMC2212486

[131] V. Ovcinnikovs , E. M. Ross , L. Petersone , N. M. Edner , F. Heuts , E. Ntavli , A. Kogimtzis , A. Kennedy , C. J. Wang , C. L. Bennett , D. M. Sansom , L. S. K. Walker , Sci. Immunol. 2019, 4, eaaw0902. 31152091 10.1126/sciimmunol.aaw0902 PMC6570622

[132] M. F. Krummel , J. P. Allison , J. Exp. Med. 1995, 182, 459. 7543139 10.1084/jem.182.2.459 PMC2192127

[133] E. Lozano , M. Dominguez‐Villar , V. Kuchroo , D. A. Hafler , J. Immunol. 2012, 188, 3869. 22427644 10.4049/jimmunol.1103627 PMC3324669

[134] C. A. Fuhrman , W. I. Yeh , H. R. Seay , P. Saikumar Lakshmi , Z. Lu , C. E. Kendal‐Wright , T. Smith , D. Masiello , J. Chang , H. S. Kuehn , K. M. Schwartz , G. L. Turk , E. B. Hill , D. C. Dwyer , D. T. Ruane , S. M. Hayes , R. H. Schwartz , J. D. Powell , M. B. Headley , J. Immunol. 2015, 195, 145.25994968

[135] J. F. Grosso , C. C. Kelleher , T. J. Harris , C. H. Maris , E. L. Hipkiss , A. De Marzo , R. Anders , G. Netto , D. Getnet , T. C. Bruno , M. V. Goldberg , D. M. Pardoll , C. G. Drake , J. Clin. Invest. 2007, 117, 3383. 17932562 10.1172/JCI31184 PMC2000807

[136] J. A. Perry , L. Shallberg , J. T. Clark , J. A. Gullicksrud , J. H. DeLong , B. B. Douglas , A. P. Hart , Z. Lanzar , K. O’Dea , C. Konradt , J. Park , J. R. Kuchroo , D. Grubaugh , A. Glatman Zaretsky , I. E. Brodsky , R. de Waal Malefyt , D. A. Christian , A. H. Sharpe , C. A. Hunter , Nat. Immunol. 2022, 23, 743. 35437326 10.1038/s41590-022-01170-w PMC9106844

[137] G. Fanelli , M. Romano , E. Nova‐Lamperti , M. Werner Sunderland , A. Nerviani , C. Scottà , M. Bombardieri , S. A. Quezada , S. H. Sacks , R. J. Noelle , C. Pitzalis , R. I. Lechler , G. Lombardi , P. D. Becker , PLoS Biol. 2021, 19, e3001199. 33901179 10.1371/journal.pbio.3001199 PMC8101994

[138] M. Vocanson , A. Rozieres , A. Hennino , G. Poyet , V. Gaillard , J. C. Rousselle , J. Benetière , M. Berard , B. Dubois , D. Kaiserlian , J. F. Nicolas , J. Allergy Clin. Immunol. 2010, 126, 280. 20624644 10.1016/j.jaci.2010.05.022

[139] Q. Chen , L. Mo , X. Cai , L. Xie , B. Zheng , S. Wang , L. Ye , C. Huang , W. Wang , Int. J. Med. Sci. 2018, 15, 666.29910670

[140] M. Löhning , A. Hutloff , T. Kallinich , H. W. Mages , K. Bonhagen , A. Radbruch , E. Hamelmann , R. A. Kroczek , J. Exp. Med. 2003, 197, 181. 12538658 10.1084/jem.20020632 PMC2193816

[141] D. Coe , S. Begom , C. Addey , M. White , J. Dyson , J. G. Chai , Cancer Immunol. Immunother. 2010, 59, 1367. 20480365 10.1007/s00262-010-0866-5 PMC11030908

[142] S. J. Muriglan , T. Ramirez‐Montagut , O. Alpdogan , T. W. Van Huystee , J. M. Eng , V. M. Hubbard , A. A. Kochman , K. H. Tjoe , C. Riccardi , P. P. Pandolfi , S. Sakaguchi , A. N. Houghton , M. R. M. Van Den Brink , J. Exp. Med. 2004, 200, 149. 15249593 10.1084/jem.20040116 PMC2212013

[143] M. Croft , T. So , W. Duan , P. Soroosh , Immunol. Rev. 2009, 229, 173. 19426222 10.1111/j.1600-065X.2009.00766.x PMC2729757

[144] B. Valzasina , C. Guiducci , H. Dislich , N. Killeen , A. D. Weinberg , M. P. Colombo , Blood 2005, 105, 2845. 15591118 10.1182/blood-2004-07-2959

[145] A. Nowak , D. Lock , P. Bacher , T. Hohnstein , K. Vogt , J. Gottfreund , P. Giehr , J. K. Polansky , B. Sawitzki , A. Kaiser , J. Walter , A. Scheffold , Front. Immunol. 2018, 9, 199. 29467769 10.3389/fimmu.2018.00199 PMC5808295

[146] M. Wolfl , J. Kuball , W. Y. Ho , H. Nguyen , T. J. Manley , M. Bleakley , P. D. Greenberg , Blood 2007, 110, 201. 17371945 10.1182/blood-2006-11-056168 PMC1896114

[147] G. Borsellino , M. Kleinewietfeld , D. Di Mitri , A. Sternjak , A. Diamantini , R. Giometto , S. Höpner , D. Centonze , G. Bernardi , M. L. Dell’Acqua , P. M. Rossini , L. Battistini , O. Rötzschke , K. Falk , Blood 2007, 110, 1225. 17449799 10.1182/blood-2006-12-064527

[148] F. Raczkowski , A. Rissiek , I. Ricklefs , M. Heiss , A. Schumacher , F. Beisner , J. Haag , A. Weiterer , L. Ulas , K. E. Steiner , A. M. Kaufmann , M. D. Leonhardt , C. Kirschning , A. Peters , N. C. Riedemann , J. L. Schultze , H. Y. Eltzschig , F. Haag , F. Koch‐Nolte , T. Magnus , PLoS One 2018, 13, e0197151. 29742141 10.1371/journal.pone.0197151 PMC5942830

[149] F. Fang , M. Yu , M. M. Cavanagh , M. Hutter , H. Kent , G. Chen , M. A. Battistone , H. Huang , F. C. Mackey , M. D. Feldman , N. Marshall , J. Ramirez , J. Fu , R. Madan , R. C. Fuhlbrigge , W. Liao , Y. Miao , B. Lin , E. M. Behrens , J. Snyder‐Cappione , P. Tu , C. M. Weyand , J. J. Goronzy , Cell Rep. 2016, 14, 1218.26832412

[150] E. Schneider , R. Winzer , A. Rissiek , I. Ricklefs , J. Meyer , P.‐H. Mertes , F. Sawitza , J. Haarmann , G. S. Kuschke , G. Sponer , A. Hanisch , S. Klawitter , L. Weidner , A. Michels , E. Richard , T. Magnus , F. Haag , F. Koch‐Nolte , G. Hartmann , J. Mattner , Nat. Commun. 2021, 12, 5911. 34625545 10.1038/s41467-021-26134-w PMC8501027

[151] K. Watanabe , A. M. Gomez , S. Kuramitsu , K. Okuma , K. Sato , K. Kobayashi , H. Hayashi , H. Tokunaga , Y. Suetsugu , A. Goto , K. Nishimura , H. Matsushita , N. Ishii , S. Nishikawa , H. Shiku , M. Harada , Blood Adv. 2023, 7, 3416.37058474

[152] T. Akimova , U. H. Beier , L. Wang , M. H. Levine , W. W. Hancock , PLoS One 2011, 6, e24226. 21918685 10.1371/journal.pone.0024226 PMC3168881

[153] S. E. Allan , S. Q. Crome , N. K. Crellin , L. Passerini , T. S. Steiner , R. Bacchetta , M. G. Roncarolo , M. K. Levings , Int. Immunol. 2007, 19, 345. 17329235 10.1093/intimm/dxm014

[154] U. Baron , S. Floess , G. Wieczorek , K. Baumann , A. Grützkau , J. Dong , A. Thiel , T. J. Boeld , P. Hoffmann , M. Edinger , I. Türbachova , A. Hamann , S. Olek , J. Huehn , Eur. J. Immunol. 2007, 37, 2378. 17694575 10.1002/eji.200737594

[155] P. Milpied , A. Renand , J. Bruneau , D. A. Mendes‐da‐Cruz , S. Jacquelin , V. Asnafi , M. T. Rubio , E. MacIntyre , Y. Lepelletier , O. Hermine , Eur. J. Immunol. 2009, 39, 1466. 19499532 10.1002/eji.200839040

[156] N. Zimmer , E. R. Trzeciak , B. Graefen , K. Satoh , A. Tuettenberg , Front. Immunol. 2022, 13, 928450. 35898500 10.3389/fimmu.2022.928450 PMC9309211

[157] F. Noyan , Y. S. Lee , K. Zimmermann , M. Hardtke‐Wolenski , R. Taubert , G. Warnecke , A. K. Knoefel , E. Schulde , S. Olek , M. P. Manns , E. Jaeckel , Eur. J. Immunol. 2014, 44, 2592. 24990119 10.1002/eji.201344381

[158] H. Wang , H. Song , A. V. Pham , L. J. Cooper , J. J. Schulze , S. Olek , D. Q. Tran , Theranostics 2019, 9, 2315. 31149046 10.7150/thno.30254 PMC6531299

[159] W. Liu , A. L. Putnam , Z. Xu‐Yu , G. L. Szot , M. R. Lee , S. Zhu , P. A. Gottlieb , P. Kapranov , T. R. Gingeras , B. Fazekas de St Groth , C. Clayberger , D. M. Soper , S. F. Ziegler , J. A. Bluestone , J. Exp. Med. 2006, 203, 1701. 16818678 10.1084/jem.20060772 PMC2118339

[160] J. D. Weaver , E. C. Stack , J. A. Buggé , C. Hu , L. McGrath , A. Mueller , M. Wong , B. Klebanov , T. Rahman , R. Kaufman , C. Fregeau , V. Spaulding , M. Priess , K. Legendre , S. Jaffe , D. Upadhyay , A. Singh , C. A. Xu , K. Krukenberg , Y. Zhang , Y. Ezzyat , D. Saddier Axe , M. R. Kuhne , M. A. Meehl , D. R. Shaffer , B. M. Weist , D. Wiederschain , F. Depis , M. Gostissa , Oncoimmunology 2022, 11, 2141007. 36352891 10.1080/2162402X.2022.2141007 PMC9639568

[161] H. Van Damme , B. Dombrecht , M. Kiss , H. Roose , E. Allen , E. Van Overmeire , D. Kancheva , L. Martens , A. Murgaski , P. M. R. Bardet , G. Blancke , M. Jans , E. Bolli , M. S. Martins , Y. Elkrim , J. Dooley , L. Boon , J. K. Schwarze , F. Tacke , K. Movahedi , N. Vandamme , B. Neyns , S. Ocak , I. Scheyltjens , L. Vereecke , F. A. Nana , P. Merchiers , D. Laoui , J. A. Van Ginderachter , J. Immunother. Cancer 2021, 9, e001749. 33589525 10.1136/jitc-2020-001749 PMC7887378

[162] S. Nagata , T. Ise , I. Pastan , J. Immunol. 2009, 182, 7518. 19494275 10.4049/jimmunol.0802230 PMC2745186

[163] B. Hou , L. Zhou , H. Wang , M. Saeed , D. Wang , Z. Xu , Y. Li , H. Yu , Adv. Mater. 2020, 32, e1907210. 32048361 10.1002/adma.201907210

[164] W. Quan , M. Ramirez , C. Taylor , F. Quan , M. Vinogradov , P. Walker , Cancer Biother. Radiopharm. 2005, 20, 11. 15778574 10.1089/cbr.2005.20.11

[165] M. D. McSweeney , L. S. L. Price , T. Wessler , E. C. Ciociola , L. B. Herity , J. A. Piscitelli , A. C. DeWalle , T. N. Harris , A. K. P. Chan , R. S. Saw , P. Hu , J. C. Jennette , M. G. Forest , Y. Cao , S. A. Montgomery , W. C. Zamboni , S. K. Lai , J. Control Release 2019, 311–312, 138. 10.1016/j.jconrel.2019.08.017 PMC6874909 31454530

[166] G. Hannon , J. Lysaght , N. J. Liptrott , A. Prina‐Mello , Adv. Sci. Weinh. 2019, 6, 1900133. 31592123 10.1002/advs.201900133 PMC6774033

[167] M. Delaval , S. Boland , B. Solhonne , M. A. Nicola , S. Mornet , A. Baeza‐Squiban , J. M. Sallenave , I. Garcia‐Verdugo , Part Fibre Toxicol. 2015, 12, 1. 25605549 10.1186/s12989-014-0078-9 PMC4318199

[168] A. A. Shvedova , J. P. Fabisiak , E. R. Kisin , A. R. Murray , J. R. Roberts , Y. Y. Tyurina , J. M. Antonini , W. H. Feng , C. Kommineni , J. Reynolds , A. Barchowsky , V. Castranova , V. E. Kagan , Am. J. Respir. Cell Mol. Biol. 2008, 38, 579. 18096873 10.1165/rcmb.2007-0255OC PMC2335338

[169] K. P. Vadalasetty , C. Lauridsen , R. M. Engberg , R. Vadalasetty , M. Kutwin , A. Chwalibog , E. Sawosz , BMC Vet. Res. 2018, 14, 1. 29291752 10.1186/s12917-017-1323-x PMC5748950

[170] J. Małaczewska , Pol. J. Vet. Sci. 2014, 17, 263. 24988852 10.2478/pjvs-2014-0037

[171] H. K. Makadia , S. J. Siegel , Polymers Basel 2011, 3, 1377. 22577513 10.3390/polym3031377 PMC3347861

[172] J. Fernandez Alarcon , M. Soliman , T. U. Lüdtke , E. Clemente , M. Dobricic , M. B. Violatto , A. Corbelli , F. Fiordaliso , C. Cordiglieri , L. Talamini , G. Sitia , S. Moya , P. Bigini , M. P. Monopoli , Nanoscale. 2023, 15, 8740. 37097471 10.1039/d3nr00685a

[173] M. R. K. Ali , M. A. Rahman , Y. Wu , T. Han , X. Peng , M. A. Mackey , D. Wang , H. J. Shin , Z. G. Chen , H. Xiao , R. Wu , Y. Tang , D. M. Shin , M. A. El‐Sayed , Proc. Natl. Acad. Sci. 2017, 114, E3110. 28356516 10.1073/pnas.1619302114 PMC5393247

[174] K. Jakic , M. Selc , F. Razga , Z. Rotter , N. Marolt , S. Smrke , P. Dular , M. Kristan , K. Stana‐Kleinschek , U. Maver , Int. J. Nanomed. 2024, 19, 4103.

[175] R. M. Crist , J. H. Grossman , A. K. Patri , S. T. Stern , M. A. Dobrovolskaia , P. P. Adiseshaiah , J. D. Clogston , S. E. McNeil , Integr. Biol. Camb. 2013, 5, 66. 22772974 10.1039/c2ib20117h PMC3499664

[176] G. Hannon , A. Prina‐Mello , Wiley Interdiscip. Rev. Nanomed. Nanobiotechnol. 2021, 13, e1738. 34254460 10.1002/wnan.1738

[177] G. Hannon , B. J. Heaton , A. Plant‐Hately , C. David , N. J. Liptrott , A. Egizabal , A. Ayerdi‐Izquierdo , N. Alvarez , O. Ibarrola , A. Arbona Celaya , A. Del Pozo Perez , N. Lazcanoiturburu , I. Luzuriaga , F. D. Zegeye , S. Zienolddiny‐Narui , A. Jacobs , A. Van Driessche , I. Nelissen , I. Abasolo , F. Andrade , N. Ventosa , E. González‐Mira , A. Carreño , A. Prina‐Mello , Nanoscale 2024, 16, 21011. 39445396 10.1039/d4nr02821j

[178] B. W. Neun , M. A. Dobrovolskaia , Methods Mol. Biol. 2018, 1682, 23. 29039090 10.1007/978-1-4939-7352-1_3

[179] N. Desai , AAPS J. 2012, 14, 282. 22407288 10.1208/s12248-012-9339-4 PMC3326161

[180] H. Xie , J. W. Smith , J. Nanobiotechnol. 2010, 8, 18. 10.1186/1477-3155-8-18 PMC2929212 20707919

[181] S. Wren , C. Minelli , Y. Pei , N. Akhtar , J. Pharm. Sci. 2020, 109, 2284. 32278922 10.1016/j.xphs.2020.04.001

[182] K. G. Reuter , J. L. Perry , D. Kim , J. C. Luft , R. Liu , J. M. DeSimone , Nano Lett. 2015, 15, 6371. 26389971 10.1021/acs.nanolett.5b01362 PMC4772408

[183] S. G. Elci , Y. Jiang , B. Yan , S. T. Kim , K. Saha , D. F. Moyano , G. Yesilbag Tonga , L. C. Jackson , V. M. Rotello , R. W. Vachet , ACS Nano 2016, 10, 5536. 27164169 10.1021/acsnano.6b02086

[184] M. C. Operti , A. Bernhardt , V. Sincari , E. Jager , S. Grimm , A. Engel , M. Hruby , C. G. Figdor , O. Tagit , Pharmaceutics 2022, 14, 276. 35214009 10.3390/pharmaceutics14020276 PMC8878443

[185] L. Rao , Y. Yuan , X. Shen , G. Yu , X. Chen , Nat. Nanotechnol. 2024, 19, 1769. 39362960 10.1038/s41565-024-01753-8

[186] Z. Kis , Pharmaceutics 2022, 14, 430. 35214162 10.3390/pharmaceutics14020430 PMC8877932

[187] H. Muramatsu , K. Lam , C. Bajusz , D. Laczko , K. Karikó , P. Schreiner , A. Martin , P. Lutwyche , J. Heyes , N. Pardi , Mol. Ther. 2022, 30, 1941. 35131437 10.1016/j.ymthe.2022.02.001 PMC8815268

[188] H. Bastian , N. Lounnas‐Mourey , P. Heimendinger , B. L. Hsu , K. H. Schreeb , C. Chapman , E. Culme‐Seymour , G. F. Atkinson , D. Cantarovich , Transl. Med. Commun. 2023, 8, 15.

[189] M. Wiesinger , D. Stoica , S. Roessner , C. Lorenz , A. Fischer , R. Atreya , C. F. Neufert , I. Atreya , A. Scheffold , B. Schuler‐Thurner , M. F. Neurath , G. Schuler , C. J. Voskens , Front. Immunol. 2017, 8, 1371. 29123521 10.3389/fimmu.2017.01371 PMC5662555

[190] M. Iizuka‐Koga , H. Nakatsukasa , M. Ito , T. Akanuma , Q. Lu , A. Yoshimura , J. Autoimmun. 2017, 83, 113. 28709726 10.1016/j.jaut.2017.07.002

[191] R. J. Miragaia , T. Gomes , A. Chomka , L. Jardine , A. Riedel , A. N. Hegazy , N. Whibley , A. Tucci , X. Chen , I. Lindeman , G. Emerton , T. Krausgruber , J. Shields , M. Haniffa , F. Powrie , S. A. Teichmann , Immunity 2019, 50, 493. 30737144 10.1016/j.immuni.2019.01.001 PMC6382439

[192] S. Kumagai , S. Koyama , K. Itahashi , T. Tanegashima , Y.-T. Lin , Y. Togashi , T. Kamada , T. Irie , G. Okumura , H. Kono , D. Ito , R. Fujii , S. Watanabe , A. Sai , S. Fukuoka , E. Sugiyama , G. Watanabe , T. Owari , H. Nishinakamura , D. Sugiyama , Y. Maeda , A. Kawazoe , H. Yukami , K. Chida , Y. Ohara , T. Yoshida , Y. Shinno , Y. Takeyasu , M. Shirasawa , K. Nakama , et al., Cancer Cell. 2022, 40, 201. 35090594 10.1016/j.ccell.2022.01.001

[193] A. Castiglioni , Y. Yang , K. Williams , A. Gogineni , R. S. Lane , A. W. Wang , J. A. Shyer , Z. Zhang , S. Mittman , A. Gutierrez , J. L. Astarita , M. Thai , J. Hung , Y. A. Yang , T. Pourmohamad , P. Himmels , M. De Simone , J. Elstrott , A.-H. Capietto , R. Cubas , Z. Modrusan , W. Sandoval , J. Ziai , S. E. Gould , W. Fu , Y. Wang , J. T. Koerber , S. Sanjabi , I. Mellman , S. J. Turley , et al., Nat. Commun. 2023, 14, 4703. 37543621 10.1038/s41467-023-40398-4 PMC10404279

[194] K. G. MacDonald , R. E. Hoeppli , Q. Huang , J. Gillies , D. S. Luciani , P. C. Orban , R. Broady , M. K. Levings , J. Clin. Invest. 2016, 126, 1413. 26999600 10.1172/JCI82771 PMC4811124

[195] A. C. Boroughs , R. C. Larson , B. D. Choi , A. A. Bouffard , L. S. Riley , E. Schiferle , A. S. Kulkarni , C. L. Cetrulo , D. Ting , B. R. Blazar , S. Demehri , M. V. Maus , JCI Insight 2019, 5, e126194. 30869654 10.1172/jci.insight.126194 PMC6538349

[196] N. A. J. Dawson , M. K. Levings , Transl Res. 2017, 187, 53. 28688236 10.1016/j.trsl.2017.06.009

[197] K. Schreeb , E. Culme‐Seymour , E. Ridha , C. Dumont , G. Atkinson , B. Hsu , P. Reinke , Kidney Int. Rep. 2022, 7, 1258. 35694562 10.1016/j.ekir.2022.03.030 PMC9174048

[198] M. M. Billingsley , N. Singh , P. Ravikumar , R. Zhang , C. H. June , M. J. Mitchell , Nano Lett. 2020, 20, 1578. 31951421 10.1021/acs.nanolett.9b04246 PMC7313236

[199] J. G. Rurik , I. Tombácz , A. Yadegari , P. O. Méndez Fernández , S. V. Shewale , L. Li , T. Kimura , O. Y. Soliman , T. E. Papp , Y. K. Tam , B. L. Mui , S. M. Albelda , E. Puré , C. H. June , H. Aghajanian , D. Weissman , H. Parhiz , J. A. Epstein , Science 2022, 375, 91. 34990237 10.1126/science.abm0594 PMC9983611

[200] R. Kitte , M. Rabel , R. Geczy , S. Park , S. Fricke , U. Koehl , U. Sandy Tretbar , Mol. Ther. Meth. Clin. Dev. 2023, 31, 101139. 10.1016/j.omtm.2023.101139 PMC10663670 38027056

[201] C. L. Bennett , J. Christie , F. Ramsdell , M. E. Brunkow , P. J. Ferguson , L. Whitesell , T. E. Kelly , F. T. Saulsbury , P. F. Chance , H. D. Ochs , Nat. Genet. 2001, 27, 20. 11137993 10.1038/83713

[202] M. Goodwin , E. Lee , U. Lakshmanan , S. Shipp , L. Froessl , F. Barzaghi , L. Passerini , M. Narula , A. Sheikali , C. M. Lee , G. Bao , C. S. Bauer , H. K. Miller , M. Garcia‐Lloret , M. J. Butte , A. Bertaina , A. Shah , M. Pavel‐Dinu , A. Hendel , M. Porteus , M. G. Roncarolo , R. Bacchetta , Sci. Adv. 2020, 6, eaaz0571. 32494707 10.1126/sciadv.aaz0571 PMC7202871

[203] Y. Honaker , N. Hubbard , Y. Xiang , L. Fisher , D. Hagin , K. Sommer , Y. Song , S. J. Yang , C. Lopez , T. Tappen , E. M. Dam , I. Khan , M. Hale , J. H. Buckner , A. M. Scharenberg , T. R. Torgerson , D. J. Rawlings , Sci. Transl. Med. 2020, 12, eaay6422. 32493794 10.1126/scitranslmed.aay6422

